# Archaeal and bacterial communities assembly and co-occurrence networks in subtropical mangrove sediments under *Spartina alterniflora* invasion

**DOI:** 10.1186/s40793-021-00377-y

**Published:** 2021-05-03

**Authors:** Weidong Chen, Donghui Wen

**Affiliations:** College of Environmental Sciences and Engineering, Peking University, Beijing, 100871 China

**Keywords:** Community assembly, Co-occurrence network, Bacterial community, Archaeal community, Mangrove, *Spartina alterniflora*

## Abstract

**Background:**

Mangrove ecosystems are vulnerable due to the exotic *Spartina alterniflora* (*S. alterniflora*) invasion in China. However, little is known about mangrove sediment microbial community assembly processes and interactions under *S. alterniflora* invasion. Here, we investigated the assembly processes and co-occurrence networks of the archaeal and bacterial communities under *S. alterniflora* invasion along the coastlines of Fujian province, southeast China.

**Results:**

Assembly of overall archaeal and bacterial communities was driven predominantly by stochastic processes, and the relative role of stochasticity was stronger for bacteria than archaea. Co-occurrence network analyses showed that the network structure of bacteria was more complex than that of the archaea. The keystone taxa often had low relative abundances (conditionally rare taxa), suggesting low abundance taxa may significantly contribute to network stability. Moreover, *S. alterniflora* invasion increased bacterial and archaeal drift process (part of stochastic processes), and improved archaeal network complexity and stability, but decreased the network complexity and stability of bacteria. This could be attributed to *S. alterniflora* invasion influenced microbial communities diversity and dispersal ability, as well as soil environmental conditions.

**Conclusions:**

This study fills a gap in the community assembly and co-occurrence patterns of both archaea and bacteria in mangrove ecosystem under *S. alterniflora* invasion. Thereby provides new insights of the plant invasion on mangrove microbial biogeographic distribution and co-occurrence network patterns.

**Supplementary Information:**

The online version contains supplementary material available at 10.1186/s40793-021-00377-y.

## Background

Mangroves, as a blue carbon reservoir, lie in special locations connecting coastal and estuarine areas [1]. The microorganisms that inhabit mangrove sediments play a critical role in the biogeochemical cycling (i.e. methane cycling, ammonia oxidation, sulfate reduction) and the deposition of heavy metals from adjacent land [2, 3]. However, Chinese mangrove ecosystems are vulnerable to the invasive species *Spartina alterniflora*, which was introduced in the 1970s and has spread throughout mangrove coastlines over the past few decades [4]. Extensive studies have showed that *S. alterniflora* invasion may alter mangrove ecosystem functions through a variety of mechanisms, such as changing plant, animal and microbial biodiversity [5, 6] and carbon or nitrogen cycling (e.g. soil N_2_O emissions) [7]. Whereas the effect of the *S. alterniflora* invasion on mangrove microbial community assembly and species interactions remains unclear.

Determining the mechanisms and processes controlling community diversity and biogeography across earth’s ecosystems is a central topic in microbial ecology [8, 9]. Niche-based and neutral-based theories constitute two important and complementary mechanisms for understanding microbial community assembly [10, 11]. Niche-based theory asserts that deterministic processes largely control the patterns of community structure. In general, deterministic processes involve nonrandom, niche-based mechanisms, including environmental filtering and various biological interactions (e.g., competition, facilitation, mutualisms, and predation) [12, 13]. Neutral theory assumes that all species or individuals are ecologically functionally equivalent, and species dynamics are controlled by stochastic processes [9, 10]. In contrast to deterministic processes, stochastic processes consider that community diversity is maintained by a few indistinguishable processes including probabilistic dispersal (e.g., random chance for colonization), random speciation and extinction, and ecological drift (e.g., random changes in organismal abundance) [8–10, 12]. Recently, it has been generally accepted that both deterministic and stochastic processes occur simultaneously in the assembly of metacommunity. A central debate currently focuses on their relative importance in controlling community structure, succession, and biogeography [12–14]. Over the past few decades, the biogeographic patterns of microbial communities have been reported for a wide range of ecosystems at different scales such as in subtropical river [15], agricultural soil [16] and wastewater treatment plants [17]. However, in the mangroves, which are essential intertidal ecosystems [18], microbial biogeographic patterns are still poorly understood. Disentangling the mangrove microbial ecological processes and interaction will promote our understanding of the mangroves ecological function.

Uncovering species coexistence in microorganism communities is an enduring challenge and large gap for microbial ecologists. Co-occurrence networks often reveal non-random co-variation patterns which may reflect community organization – such as direct interactions [19] or shared guilds or niches, and provide a tool for investigating ecological concepts which are difficult to assess in microbial communities [20–22]. Under this approach, interactive taxa are linked together either positively or negatively indicating mutualistic or antagonistic co-occurrence patterns. Network analysis has been used to explore microbial interactions and/or symbiotic patterns among different microbial taxa in various environments [20–22]. Furthermore, by identifying the most connected microbial populations or analyzing the effects of nodes and linkages of different methods, network analysis could also identify keystone species that may have the greatest impact on microbial community structure and potential functions [19–23]. A previous study showed that the core microbes with abundant and ubiquitous characters in mangroves were mostly assigned to Gammaproteobacteria, Deltaproteobacteria, Chloroflexi and Euryarchaeota [24]. Meanwhile, the role of rare species in microbial networks remain still unclear. A better understanding of the mechanisms that influence highly connected taxa composition and structure may provide an insight into the underlying response of the whole community [21]. Currently, it is well recognized that both bacteria and archaea are abundant and critical in mangrove, whereas most studies explored mangrove plant, animal and microbial diversity, greenhouse gas emissions and climate change [18, 24]. And these studies focused largely on single taxonomic group [6, 24]. There remains knowledge gap on comparison of ecological processes and species co-occurrence network of both bacterial and archaeal communities in mangrove ecosystems.

The mangrove ecosystem across the coastlines in Fujian province, southeast China is occupied by *S. alterniflora*, which make it an ideal system to address the effect of plant invasion on microbial ecological processes and species interaction. Here, we characterized the mangrove microbiome using 48 sediment samples (each 12 samples from 4 different types of vegetation zones including mangrove, ecotone, cordgrass, and mudflat) from four mangrove regions (Fig. S1). We focus on comparing the ecological distribution patterns and co-occurrence of bacteria and archaea, meanwhile, explore *S. alterniflora* invasion on the community assembly and microbial interaction. Considering the difference of diversity, abundance, dispersal ability and environmental tolerance of archaea and bacteria [25], we firstly hypothesize that the community assembly and co-occurrence network patterns are different between these two groups. In addressing this hypothesis, we compare the overall archaeal and bacterial community assembly and co-occurrence patterns. Furthermore, the *S. alterniflora* invasion may affect microbial community and sediment physicochemical factors [6, 7], thus we secondly hypothesize that *S. alterniflora* will change the relative importance of deterministic and stochastic processes and microbial co-occurrence relationships. In addressing this hypothesis, we separately compare the community assembly mechanisms and network complexity under four different types of vegetation zones (mangrove, ecotone, cordgrass, and mudflat). The aims of the present study were to: 1) compare the difference of bacterial and archaeal community assembly and co-occurrence networks; and 2) evaluate the influence of *S. alterniflora* invasion on bacterial and archaeal community assembly and co-occurrence networks.

## Results

### Microbial community composition and alpha-diversity

Rarefaction curves for a combined set of 48 samples showed that the archaeal and bacterial communities data tended to approach saturation. Furthermore, the truncated Preston log-normal model showed that our sampling found 84.77–86.51% of the archaeal OTUs and 94.86–95.18% of the bacterial OTUs (Fig. S2). These results indicated that the majority of the microbial taxa had been recovered from the studied metacommunity. A total of 6836 archaeal and 31,639 bacterial OTUs were identified from 332,304 archaeal and 2,081,856 bacterial high-quality sequences at 97% identity level, and all microbial taxa were divided into five categories (no OTU was detected as moderate taxa, Table S1). We found that bacteria showed higher alpha diversity (richness and shannon-wiener index) than archaea (Fig. S3, Tukey’s HSD test). *S. alterniflora* invasion altered microbial alpha diversity although no significant differences were found among different vegetation zones of archaea and bacteria (Fig. S4, Tukey’s HSD test). The most three abundant archaeal phyla among four different types of vegetation zones were Thaumarchaeota (54.47%), Nanoarchaeaeota (20.57%) and Crenarchaeota (18.20%). The most three abundant bacterial phyla were Proteobacteria (49.61%), Chloroflexi (11.98%) and Bacteroidetes (9.85%) (Fig. S5).

### Ecological processes controlling overall mangrove microbial community assembly

**Fig. 1:**
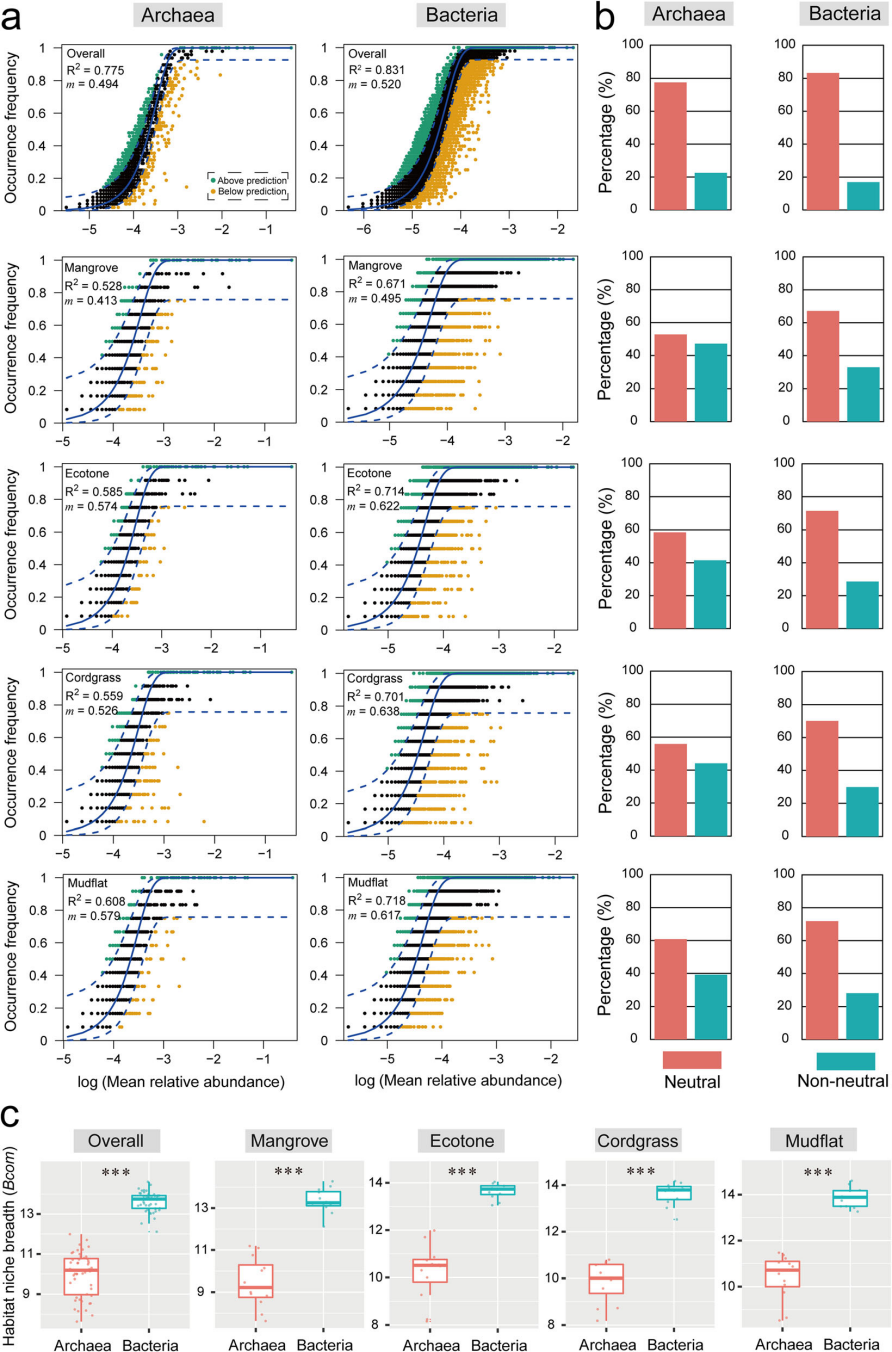
Fit of the neutral community model (NCM) of community assembly and niche breadth for archaeal and bacterial communities. **a**, **b**: Fit of the neutral community model (NCM) of community assembly. The OTUs that occurred more frequently than predicted by the model are shown in green, while those occurred less frequently than predicted are shown in orange. Blue dashed lines represent 95% confidence intervals around the model prediction and the OTUs fall within the confidence intervals were considered as neutrally distributed. *m* indicates the estimated migration rate and *R*^*2*^ indicates the fit to the neutral model. Neutral processes are the part within 95% confidence interval (red) while non-neutral are the parts including above and below prediction (dark green). **c**: Box plots illustrating standardized Levins’ niche breadth of bacteria and archaea at the community level (*Bcom*) in overall and four different types of vegetation zones. ***, *P* < 0.001 (Tukey’s HSD test). “Overall” is the combined data of four different types of vegetation zones, which represents the overall microorganisms in mangrove ecosystem

The Sloan neutral model showed that the overall archaeal and bacterial communities were driven predominantly by stochastic processes, with the *R*^*2*^ value were 0.775 and 0.831 for overall archaea and bacteria, respectively, and larger explained community variance for bacteria was observed (Fig. 1a, b). The estimated migration rate (*m*), a measure of the influence of dispersal on community composition, were higher in overall bacteria (0.520) than in overall archaea (0.494). Both the *R*^*2*^ and estimated migration rate (*m*) indicated a stronger effect of dispersal limitation on archaea than bacteria. The community-level habitat niche breadths (*Bcom*) were estimated to reveal the contributions of deterministic and stochastic processes. Niche breadth (*Bcom*) of bacteria was much greater than that of archaea in overall mangrove ecosystem and four different types of vegetation zones (Tukey’s HSD test, *P* < 0.001, Fig. 1c).

**Fig. 2:**
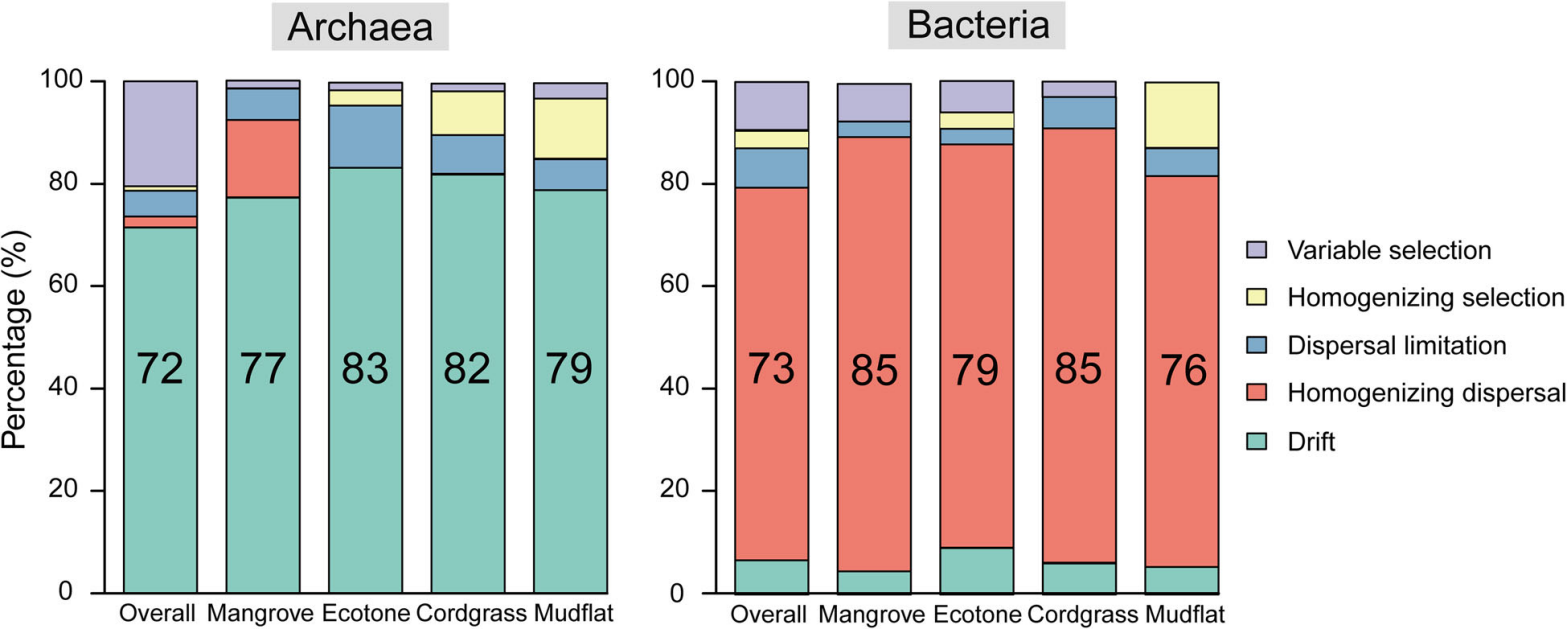
Null model analysis revealing the assembly mechanism of the archaeal and bacterial communities of mangrove sediments. The number indicate the contributions of different processes

The null model indicated that the differential action of ecological processes may promote different biogeographic patterns in archaeal and bacterial assemblages. However, the stochastic processes (sum of dispersal limitation, homogenizing dispersal, and drift) explained a higher proportion of the archaeal and bacterial communities (including the overall mangrove ecosystem and four different types of vegetation zones) variation than deterministic processes (Fig. 2), which supported the results of the neutral community model. These results suggested that stochasticity was more important than determinism in influencing mangrove microbial community. The stochastic processes accounted for 79 and 87% of the community assembly in the overall archaea and bacteria, respectively, and bacteria were more controlled by stochasticity than archaea (Fig. 2). Drift and homogenizing dispersal were the most important processes, accounting for 72 and 73% of the archaeal and bacterial communities variation, respectively.

### *Spartina alterniflora* invasion changed mangrove microbial community assembly

To determine the effect of *Spartina alterniflora* invasion on the archaeal and bacterial community assembly, the neutral community model (NCM) and null model were also used with the datasets from four different vegetation zones for archaea and bacteria. The NCM showed that *S. alterniflora* invasion changed the relative contribution of ecological processes controlling microbial community assembly (Fig. 1a, b). The values of *R*^*2*^ and immigration rate (*m*) distribution showed same pattern for archaeal and bacterial subcommunities: Mudflat (*R*^*2*^ = 0.608 and *m* = 0.579 for archaea; *R*^*2*^ = 0.718 and *m* = 0.617 for bacteria) > Ecotone (*R*^*2*^ = 0.585 and *m* = 0.574 for archaea; *R*^*2*^ = 0.714 and *m* = 0.622 for bacteria) > Cordgrass (*R*^*2*^ = 0.559 and *m* = 0.526 for archaea; *R*^*2*^ = 0.701 and *m* = 0.638 for bacteria) > Mangrove (*R*^*2*^ = 0.528 and *m* = 0.413 for archaea; *R*^*2*^ = 0.671 and *m* = 0.495 for bacteria). All of the four bacterial subcommunities fitted better to NCM than four archaeal subcommunities. The null model also suggested that invasion changed the relative contribution of microbial community assembly with different degree for archaea and bacteria. In the archaea, stochastic processes explained large subcommunities variation among four different vegetation zones with the following pattern: mangrove (98%) > ecotone (95%) > cordgrass (89%) > mudflat (85%). The bacteria showed the following pattern: cordgrass (97%) > mangrove (92%) > ecotone (91%) > mudflat (87%) (Fig. 2). In general, we found that *S. alterniflora* invasion increased bacterial and archaeal drift process.

### Overall mangrove ecosystem’ microbial network co-existence patterns

**Fig. 3:**
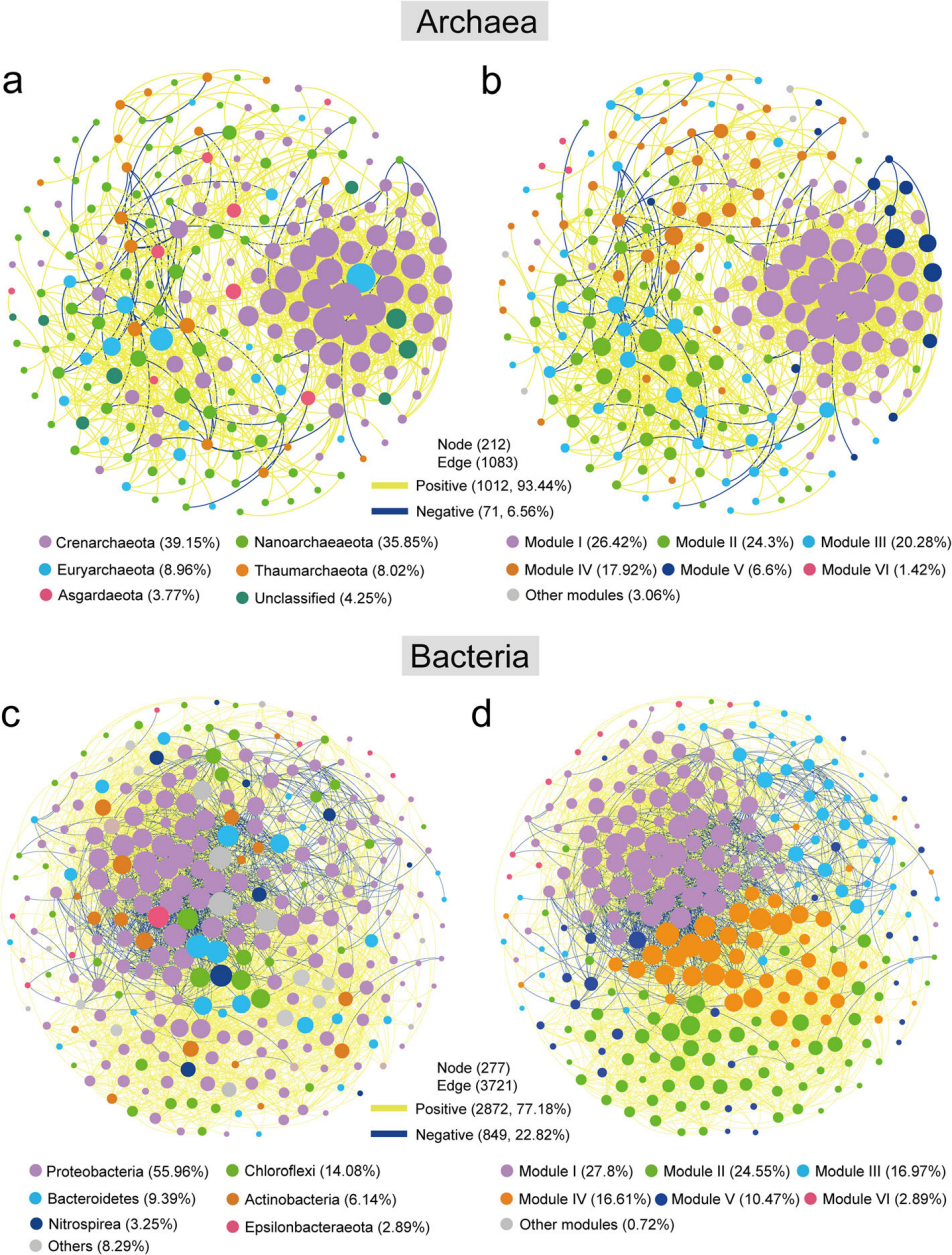
Overall co-occurrence networks of the archaeal and bacterial communities based on pairwise Spearman’s correlations between OTUs. The nodes were colored according to different types of phylums (**a**, **c**) and modularity classes (**b**, **d**), respectively. A connection stands for a strong (Spearman r > 0.6 or r < −0.6) and significant (*P*-value < 0.01) correlation. For each panel, the size of each node is proportional to the number of connections (i.e. degree). The yellow and blue edges indicate negative and positive interactions between two individual nodes, respectively

The correlation-based network consisted of 212 nodes (OTUs) with 1083 edges (correlations) for the archaea, and 277 nodes with 3721 edges for the bacteria (Table 1). Overall, taxa tended to co-occur (positive correlations, yellow lines) rather than co-exclude (negative correlations, blue lines); positive correlations accounted for 93.44 and 77.18% of the potential interactions in archaeal and bacterial networks, respectively, whereas negative correlations were 6.56 and 22.82% interactions for archaeal and bacterial co-existence patterns (Fig. 3). When considering all correlations, the links between bacteria were more complex than those between archaea (Fig. 3, Table 1), indicating that potential interactions are stronger in bacterial networks. For the archaeal network, Crenarchaeota (39.15%), Nanoarchaeaeota (35.85%), Euryarchaeaeota (8.96%), Thaumarchaeaeota (8.02%), and Asgardaeota (3.77%) mainly occupied the nodes (Fig. 3a). Nodes in bacterial network mainly belonged to Proteobacteria (55.96%), Chloroflexi (14.08%), Bacteroidetes (9.39%), Actinobacteria (6.14%), Nitrospirea (3.25%), and Epsilonbacteraeota (2.89%) (Fig. 3c). Furthermore, a module is defined as a group of OTUs that are linked more tightly together. Here, both the entire archaeal and bacterial networks were clearly parsed into 6 major modules, of which modules I, II, and III accounted for 26.42, 24.3, and 20.28% of the whole archaeal network, respectively (Fig. 3b), and modules I and II accounted for 27.8 and 24.55% of the whole bacterial network, respectively (Fig. 3d).

**Table 1:** Topological properties of the empirical species-species co-occurrence networks of bacterial and archaeal communities and their associated random network

Network properties		Archaea					Bacteria				
		Overall	Mangrove	Ecotone	Cordgrass	Mudflat	Overall	Mangrove	Ecotone	Cordgrass	Mudflat
Empirical networks	No. of original OTUs	300	300	300	300	300	300	300	300	300	300
	Network size (nodes)	212	293	295	293	289	277	296	295	294	295
	Sequence number	272,449	67,334	70,351	66,687	70,444	855,275	218,993	225,426	216,593	224,045
	Links	1083	1700	2959	1824	2477	3721	4904	4298	3224	3952
	Degree	10.217	11.604	13.281	12.451	17.142	26.866	33.135	29.139	21.932	26.793
	Average clustering coefficient	0.504	0.39	0.377	0.399	0.46	0.544	0.56	0.527	0.468	0.528
	Diameter	9	8	9	9	8	7	8	6	7	7
	Density	0.048	0.04	0.045	0.043	0.06	0.097	0.112	0.099	0.075	0.091
	Average path length	3.499	3.258	3.22	3.357	3.024	2.674	2.654	2.748	2.867	2.827
	Modularity	0.52	0.544	0.512	0.53	0.549	0.39	0.382	0.399	0.455	0.419
	*R*^*2*^ of powerlaw	0.87	0.383	0.607	0.44	0.459	0.659	0.338	0.439	0.352	0.519
Random networks	Average clustering coefficient	0.048 (± 0.040)	0.040 (± 0.002)	0.068 (± 0.002)	0.042 (± 0.002)	0.060 (± 0.002)	0.097 (± 0.002)	0.112 (± 0.001)	0.099 (± 0.001)	0.075 (± 0.002)	0.091 (± 0.001)
	Average path length	2.554 (± 0.005)	2.583 (± 0.003)	2.169 (± 0.002)	2.526 (± 0.002)	2.280 (± 0.002)	1.968 (± 0.002)	1.909 (± 0.001)	1.951 (± 0.001)	2.104 (± 0.002)	1.988 (± 0.002)
	Modularity	0.245 (± 0.011)	0.228 (± 0.008)	0.161 (± 0.006)	0.218 (± 0.008)	0.178 (± 0.007)	0.130 (± 0.005)	0.112 (± 0.005)	0.123 (± 0.005)	0.151 (± 0.006)	0.131 (± 0.005)

Random network was generated by rewiring all of the links with the same numbers of nodes and edges to the real network

Modularity > 0.4 suggests that the network has a modular structure

The number in the brackets indicates the standard deviation of mean topological properties values of the 1000 Erdös-Rényi random networks

**Fig. 4:**
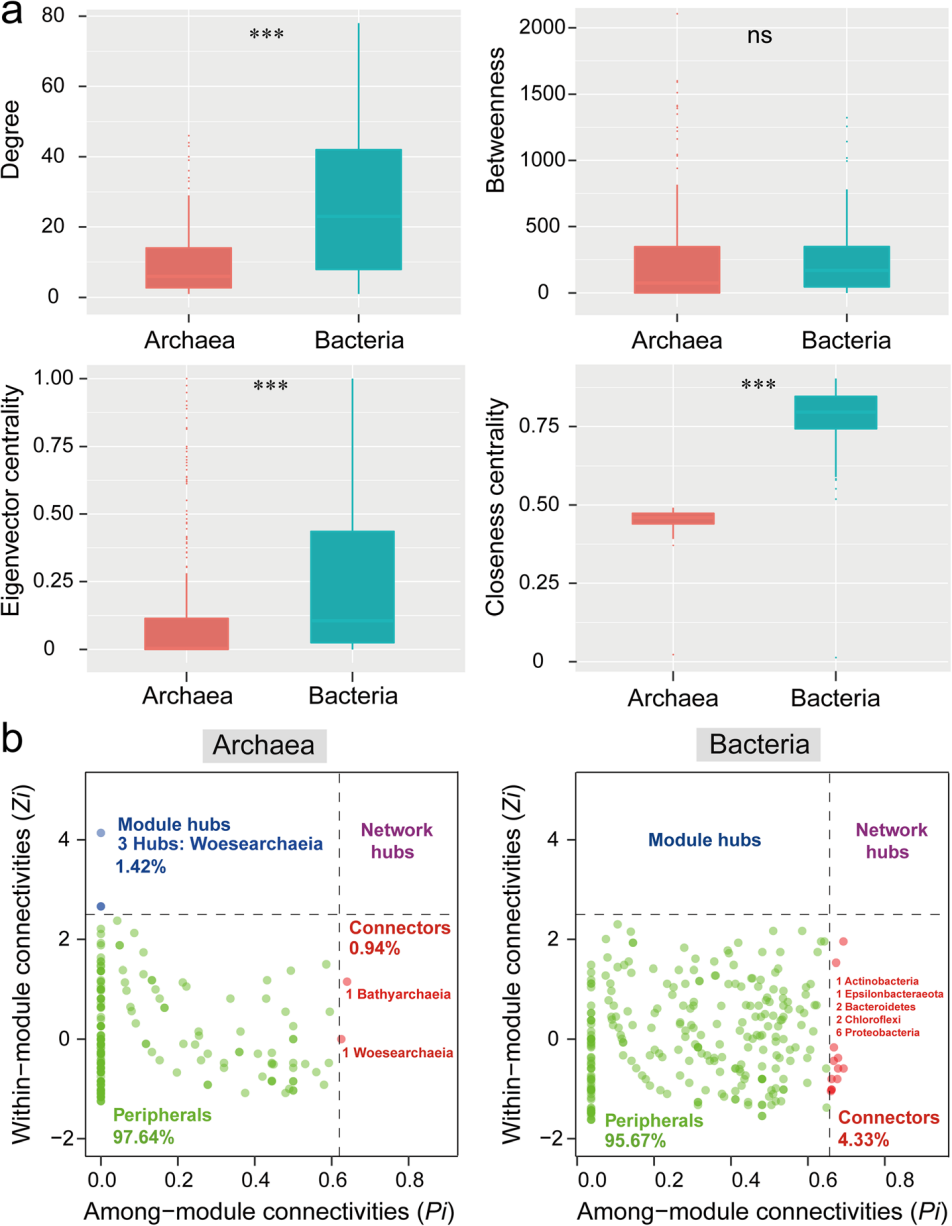
Co-occurrence network of archaeal and bacterial OTUs based on node features of the network (**a**) and Zi-Pi plot (**b**). **a**: Degree (top left); betweenness (top right); eigenvector centrality (bottom left); and closeness (bottom right). ns: not significant; *** *P* < 0.001. **b**: Zi-Pi plot showing the distribution of archaeal and bacterial OTUs based on their topological roles. Each symbol represents an OTU. The topological role of each OTU was determined according to the scatter plot of within-module connectivity (Zi) and among-module connectivity (Pi). (i) network hubs: nodes with Zi > 2.5 and Pi > 0.62; (ii) module hubs: nodes with Zi > 2.5 and Pi ≤0.62; (iii) connectors: nodes with Zi ≤ 2.5 and Pi > 0.62; and (iv) peripheral nodes: nodes with Zi ≤ 2.5 and Pi ≤0.62

The integrated network degrees were distributed according to a power-law distribution in both archaea and bacteria, indicating a scale-free distribution and non-random co-occurrence pattern (Fig. S6). We calculated a set of network-level topological features, and found that values of the degree, closeness centrality, and eigenvector centrality in bacteria were significantly higher than those in archaea (Fig. 4a, Table 1). Furthermore, the average clustering coefficients were higher in the bacterial network than that of archaea (including the overall and four different types’ vegetation zones of archaea and bacteria), suggesting that bacterial OTUs were more interconnected (Table 1). The average path length and diameter were lower in the bacterial network, revealing closer relationships among bacterial communities (including the overall and four different types’ vegetation zones of archaea and bacteria). Random networks were generated with the same nodes and edges in each compartment to confirm that the empirical networks were non-random. Details describing the constructed co-occurrence networks can be found in Table 1.

Zi-Pi plot showed that Woesearchaeia and Proteobacteria phyla were the most prominent keystone taxa for archaea and bacteria, respectively. In the co-occurrence networks, 5 archaeal OTUs and 12 bacterial OTUs were defined as keystone taxa, and Woesearchaeia and Proteobacteria phyla accounted for 80 and 50% of all module hubs and connectors (Fig. 4b; Table S2). In archaea, the keystone species include taxa from the classes Woesearchaeia (Nanoarchaeaeota, 4 OTUs) and Bathyachaeia (Crenarchaeota, 1 OTU). In bacteria, the keystone taxa include taxa from the classes Gammaproteobacteria (Proteobacteria, 3 OTUs), Actinobacteria (Actinobacteria, 1 OTU), Alphaproteobacteria (Proteobacteria, 3 OTUs), Anaerolineae (Chloroflexi, 2 OTUs), Bacteroidia (Bacteroidetes, 2 OTUs), and Campylobacteria (Epsilonbacteraeota, 1 OTU). Keystone taxa spanned a range of relative abundances (0.06 to 1.43% for archaea and 0.05 to 0.38% for bacteria). Over half of the keystone taxa (9 of 17 OTUs for both archaea and bacteria) had low relative abundance (0.05 to 0.10%). All of the 17 OTUs were conditionally rare taxa (Table S2).

### *Spartina alterniflora* invasion influenced microbial network complexity and stability

**Fig. 5:**
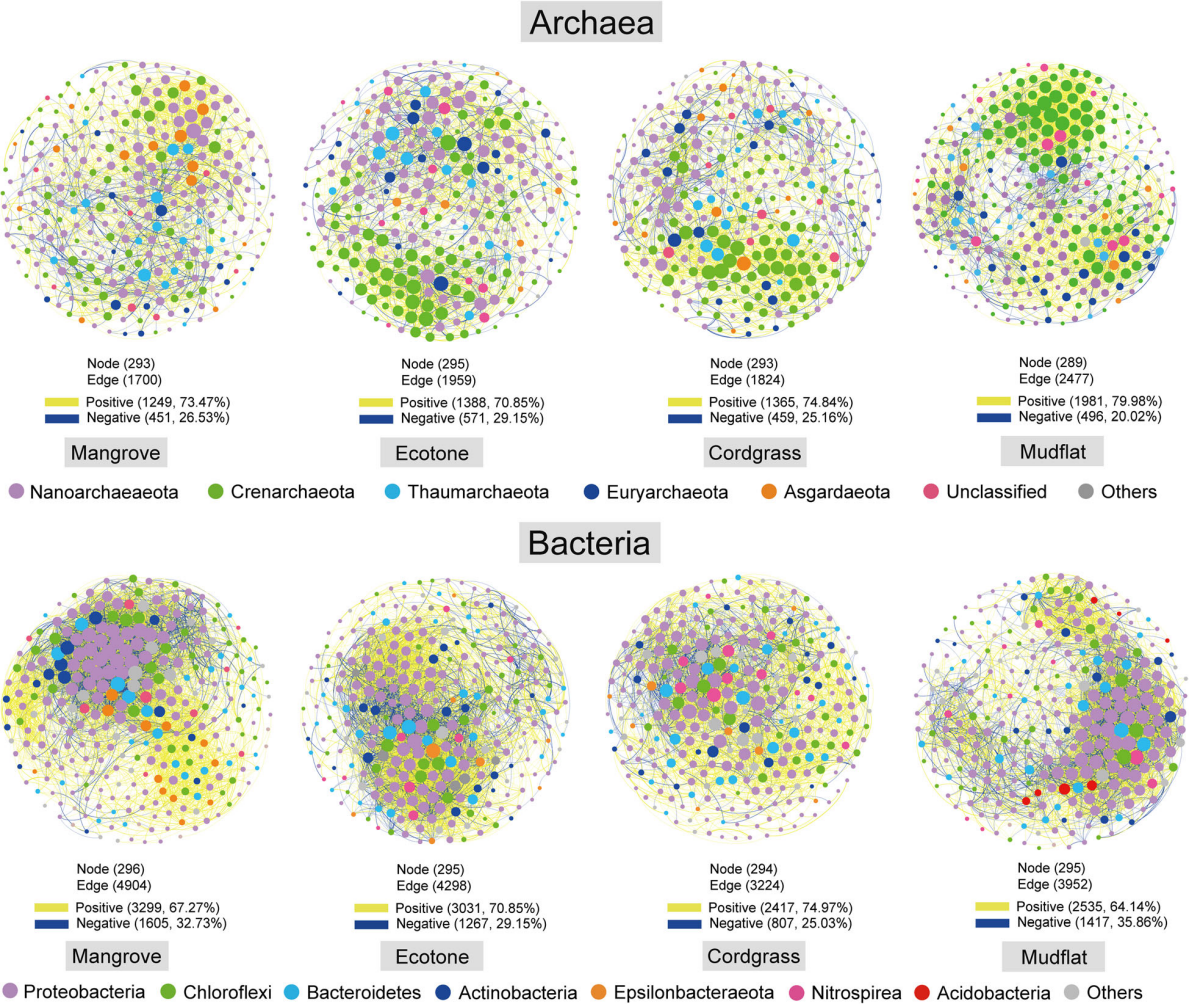
The co-occurrence patterns of mangrove sediments’ archaeal and bacterial subcommunities among four different types of vegetation zones. The size of nodes is proportional to the link numbers of each nodes. The lines between each pair of nodes represent positive (in yellow) and negative (in blue) interactions with strong (Spearman r > 0.6 or r < − 0.6) and significant (*P*-value < 0.01) correlation. Only the 300 main OTUs were included in the analysis

To identify the effect of *S. alterniflora* invasion on potential microbe-microbe interactions, we constructed four archaeal and bacterial co-occurrence networks among four different types of vegetation zones (Fig. 5). The effects of invasion on the archaeal networks differed profoundly from bacterial networks. Invasion increased archaeal network complexity and stability, but decreased the network complexity and stability of bacteria. Multiple network topological metrics (e.g. links, degree, and average clustering coefficient) consistently supported the different effect of invasion on the archaeal and bacterial co-occurrence patterns (Fig. 5, Table 1). Although the selected network size (nodes) were similar among distinct vegetation zones, the connectivity (links) of archaeal and bacterial networks were different. In the archaeal networks, the subcommunity in mudflat formed largest networks’ connections (links), followed by ecotone, cordgrass, and mangrove vegetation zones. Whereas in the bacterial networks, the network complexity showed the following trend: mangrove > ecotone > mudflat > cordgrass. The complexity of the networks was also reflected by the degree, which showed the same trend with links in the archaeal and bacterial networks (Fig. 5, Table 1). Overall, positive correlations accounted for 70–80 and 64%–75% of the potential interactions in archaeal and bacterial networks among four different vegetation zones, respectively, which were higher than negative correlations.

We compared unique node-level topological features of four subcommunities from the different vegetation zones. The network topological parameters such as betweenness centrality and closeness centrality did not differ significantly among four different vegetation zones of archaeal and bacterial subcommunities (Fig. S7). However, the degree value in the mudflat was significantly highest among four archaeal subcommunities. And in bacterial subcommunities, the ecotone showed the highest degree. Furthermore, eigenvector centrality varied significantly among different vegetations’ sediment (Tukey’s HSD test, *P* < 0.001, Fig. S7).

We found that *S. alterniflora* invasion changed keystone taxa of archaeal and bacterial subcommunities (Fig. S8-S9; Table S3-S4). In the archaeal subcommunities, a total of 82 OTUs were identified as keystone species, including the members from mangrove (28 OTUs), ecotone (26 OTUs), cordgrass (13 OTUs), and mudflat (15 OTUs) (Fig. S8; Table S3). Furthermore, in the bacterial subcommunities, 66 OTUs were considered as keystone taxa including 11 OTUs in mangrove, 11 OTUs in ecotone, 15 OTUs in cordgrass, and 29 OTUs in mudflat (Fig. S9; Table S4). Almost all of the keystone taxa were module hubs and connectors, and only one network hub was detected in all of the constructed archaeal and bacterial networks. Among four different vegetation zones, the most prominent keystone taxa in the archaeal networks were from the classes Woesearchaeia and Bathyarchaeia, and the major keystone taxa in the bacterial networks were Proteobacteria. Most of the keystone taxa were conditionally rare taxa and always rare taxa (Table S3-S4). Interestingly, 5 archaeal OTUs from Woesearchaeia and Bathyarchaeia (i.e. OTU_20 and OTU_131) and 4 bacterial OTUs from Proteobacteria and Gemmatimonadetes (i.e. OTU_18 and OTU_156) were simultaneously detected in different vegetation zones, indicating that these OTUs are important in different vegetations’ ecosystem (Table S3-S4).

To determine the effect of *S. alterniflora* invasion on the robustness of the archaeal and bacterial networks, a natural connectivity analysis was carried out among four different types of vegetation zones. In the archaeal subcommunities, the natural connectivity in the mudflat network was higher than that of mangrove, ecotone, and cordgrass vegetation zones, whereas in the bacterial subcommunities, we found the greatest natural connectivity in the mangrove vegetation zone, followed by ecotone, mudflat, and cordgrass, indicating that *S. alterniflora* invasion showed different effects on bacterial and archaeal network stability (Fig. S10).

## Discussion

To date, most studies of the mechanisms governing biogeographic patterns have focused on bacteria and microeukaryotes in soil [16], reservoirs [26] and river [15] ecosystems. This study provides a simultaneous analysis in the community assembly and co-occurrence patterns of archaea and bacteria in mangrove ecosystem, and provides novel insights for the effect of *S. alterniflora* invasion on microbial ecological processes/co-occurrence patterns.

### Similar community assembly mechanisms of overall mangrove bacteria and archaea

The neutral community model (NCM) and null model are two valid approaches for inferring community assembly, and have been successfully applied to a wide range of ecosystems [15–17, 26]. Here, NCM estimated a major part of the overall archaeal (*R*^*2*^ = 0.775) and bacterial (*R*^*2*^ = 0.831) community variation. The null model explained large archaeal (79%) and bacterial (87%) community assembly. Both of the two approaches indicated that stochastic processes (such as stochastic births, deaths, and immigration) played more important roles than deterministic processes in community assembly, and stochasticity were stronger in shaping overall bacteria than archaea (Figs. 1–2). Several studies in distinct ecosystems also revealed similar results to our finding recently, which including seven-year dynamics of testate amoeba communities in subtropical reservoirs [26], global bacterial communities in wastewater treatment plants [17], and microeukaryotes in river [15].

Previous studies have reported community assembly mechanisms of bacteria/prokaryotes and protists/microeukaryotes in marine, lake and paddy soil [27–29], however, the assembly of archaea and bacteria which share similar cell size and structure, approximately, were not previously analysed, yet they have potential difference in altered ecosystems such as invaded mangroves. The degree of stochasticity was also confirmed by the migration rates (*m*) values. Here, NCM showed that the archaea had lower *m* value compared with bacteria, suggesting that the archaea may experience more serious dispersal limitation (Fig. 1a), which caused higher stochasticity in bacteria. In addition, null model showed that heterogeneous dispersal was more important in structuring bacterial than archaeal communities (Fig. 2). The potential reason of the difference is that bacteria can be more dispersed, and they exhibit a broader range of physiologies than that of archaea, thus are easier to be successful colonists of sediment environment [30]. Therefore the more abundant and diverse bacteria are expected to be distributed more thoroughly than archaea. Indeed, we detected that bacteria exhibited significantly wider community-level habitat niche breadths than archaea (Fig. 1c), indicating that bacteria had stronger environmental tolerance or metabolic plasticity, thus showed more widely distributed pattern. This finding was in agreement with previous study which reported that habitat generalists with wider niche breadths were less influenced by environmental factors [31]. Our results also suggested that the wider niche breadth of bacteria might imply greater metabolic plasticity and higher community size than archaea. According to the sequencing results, the bacterial community showed higher alpha diversity than archaeal community (Table S1, Fig. S3). Many studies have reported that bacteria are much more abundant and diverse than archaea [32, 33]. For example, a study conducted in Chinese marginal seas surface sediment found that benthic bacteria were numerically dominant relative to archaea [33], which supported our finding. Furthermore, previous study reported that the dynamics of distinct microbial groups were constrained by different environmental variables [34]. For example, Wei and colleagues compared the archaeal and bacterial community features in bulk soils under different vegetation covers and found that similar edaphic factors showed nearly opposite effects to the two domains [34]. Their study also supported that archaea showed more niche limitation and less widely distributed than bacteria [34].

Null model analysis indicated that drift play larger role in structuring archaea than bacteria in the mangrove ecosystem, since drift tends to be more important when selection is weak and the local community size is small [12]. Ecological drift is a central concept in community ecology, which considered as stochastic changes with respect to species identity in the relative abundances of different species within a community over time due to the inherent random processes of birth, death, and reproduction [8, 14]. One study has showed that random birth and death were more important in shaping the communities with smaller population size, thus leading to increased relative importance of drift [28]. The stronger relative importance of drift in shaping archaea than bacteria suggested that the lower relative abundance and diversity might also contribute to the stochasticity of archaea (Fig. 2, Fig. S3). Another study also found a larger importance of drift in determining five data-sets of aquatic bacteria when compared with phytoplankton from freshwater / brackish habitats [35]. Differential adaptations to environmental condition in distinct domain of microbial communities (i.e. protists and prokaryotes; archaea and bacteria) may cause these differences [27].

### Differences between overall mangrove archaeal and bacterial networks

Microbial network analysis can improve our perspectives on complex interaction webs and ecological processes beyond microbial community richness and composition [36]. In this study, for the first time, we used network analysis to explore the interactions for bacteria and archaea of the mangrove ecosystem. Our result showed that the network structure of bacteria was more complex than archaea (Figs. 4-5, Table 1). The first potential reason was that bacteria had higher richness/shannon-wiener index than archaea (Fig. S3) in the studied mangrove ecosystem, thus caused more complex species’ interaction. A previous study which focused on the co-occurrence networks in a mountain ecosystem also found that low bacterial diversity reduced network complexity, which supported our finding [37]. It could also be distinct environmental factors has different effects in archaea and bacteria. Indeed, studies has showed that eukaryotic plankton co-occurrence networks were influenced by distinct environmental factors (i.e. pH, total nitrogen, and temperature) in reservoirs [38], and salinity could change bacterial co-occurrence network complexity in Tibetan Plateau lakes [39]. Santolini and colleagues revealed that complex networks with greater connectivity are more robust to environmental perturbations than simple networks with lower connectivity [40]. In general, a more complex network structure may indicate more stable co-existence patterns and higher efficiency of resource transfer. In this sense, our study confirmed that the bacterial community was more resilient to environmental stresses as different taxa could complement each other.

The network topology can be used to identify important network nodes and edges, and perform network comparison. For example, node degree can reflect the number of direct connections for a specific OTU; the closeness centrality value reflects how quickly information spreads from a given node to other reachable nodes; betweenness centrality of a node indicates the potential impacts of one species on the co-occurrences of other nodes [20, 41]. Our results found that bacteria had higher degree, eigenvector, and closeness centralities compared to archaea (Fig. 4a), indicating bacteria was more inter-connected. This observation might be because bacteria had higher diversity and ecological niche (Fig. 1c, Fig. S3), which kept bacteria has stronger buffer against the environmental disturbance. Furthermore, in this study, the positive associations outnumbered mutual exclusions in archaea (93.44% versus 6.56%) and bacteria (77.18% versus 22.82%), revealing that positive effect (i.e. mutualism and/or syntrophy, which two species exchange metabolic products to the benefit of both) exhibited a more important role than negative effect (i.e. predator-prey relationships, host-parasite relationships and/or competition between microorganisms) in studied mangrove ecosystem. Similar with the global oceanic plankton interactome conducted by *Tara* Oceans project, which found the strong role of positive correlation among viruses, prokaryotes, microbial eukaryotes, phytoplankton and zooplankton [42], indicating that microorganism tend to promote their growth.

Module which indicates similar ecological characteristics has been studied widely in microbial networks [23, 43]. Modularity in an ecological community may reflect biotic interaction and phylogenetic clustering of closely connected species [43]. Here, the modularity of the archaeal empirical co-occurrence network (0.512–0.549) showed higher value than that of bacteria (0.382–0.455) among overall archaeal and bacterial communities and four different subcommunities of vegetation zones (Table 1), indicating that the populations within the archaeal communities may have more similar modular structure [44].

### Potential keystone taxa of mangrove archaea and bacteria

Keystone taxa have been frequently referred to as “ecosystem engineers” owing to their large influence in the community, which have been reported before in various biomes, including terrestrial, aquatic and human microbiomes [21] but not in mangrove ecosystem. Here, we found that all of the 5 archaeal and 12 bacterial keystone taxa were belonged to conditionally rare taxa (CRT) (Table S2), suggesting that CRT play an important role in maintaining the stability of mangrove archaeal and bacterial networks structure [43]. The archaeal keystone OTUs belonged to the class Woesearchaeia and Bathyarchaeia (Fig. 4b; Table S2), which were discovered universal archaeal groups and distributed worldwide in anoxic marine sediments, mangrove sediments and estuarine sediments [45, 46]. Both of the two groups in mangrove ecosystems have a potential for sulfate reduction, ammonia oxidation, and organic matter decomposition [45, 47]. Meanwhile, a metagenomic survey revealed that Bathyarchaeota had metabolic capacities for acetogenesis and protein degradation in estuarine organic-rich regimes [47]. These two groups can provide new microbial biogeochemical insights on the carbon and nutrient flow in mangrove ecosystem. Furthermore, the bacterial phyla Proteobacteria, Actinobacteria, Chloroflexi, Bacteroidetes and Epsilonbacteraeota also play important role in mangrove as keystone taxa. For example, the metabolic versatility of Chloroflexi could provide a competitive advantage for surviving in fluctuating environments like mangrove ecosystem, which located in a buffer zone connecting land and ocean [48]. Proteobacteria is ubiquitous in marine environments and plays important roles in the nitrogen fixation and nutrient cycling [49]. Our study indicates that Proteobacteria as keystone taxa might be important in the nitrogen fixation and/or nutrient cycling in mangrove wetland. Increasing evidence in different habitats have shown the importance of rare and less abundant species in microbial networks [36], and their removal can cause a dramatic shift in microbiome structure and functioning. Thus conditionally rare or less abundant species should be paid more attention in the study of maintaining ecosystem function. The identification of keystone taxa could provide essential information for developing strategies to manipulate the function of microbiome and promote sustainable development of mangrove ecosystems. Nonetheless, co-occurrence networks do not always effectively predict actual classical ecological networks, thus there are still some limitations to the present approach. The omics-based profiling and culture-dependent approaches are needed to further test and understand the potential synergistic/syntrophic relationship [21, 50].

### *Spartina alterniflora* invasion changed bacterial and archaeal community assembly and network complexity and stability

*S. alterniflora* have been aggressive invaders of coastal habitats worldwide. Whereas most studies focused on *S. alterniflora* invasion altered the community abundance and diversity of related functional microorganisms, and affected C, N, and S cycles [7, 51]. The impact of invasion on community assembly and network structure was poorly understood. This study filled in this gap and observed that *S. alterniflora* changed microbial community assembly and network complexity and stability.

We speculated that there were several possible pathways by which exotic *S. alterniflora* invasion might have changed bacterial and archaeal communities assembly (Figs. 1, 2). First, *S. alterniflora* invasion could influence microbial composition and diversity (Fig. S5), because different archaeal and bacterial taxa had specific ability for dispersal [25], thus *S. alterniflora* invasion changed the contribution of stochasticity in shaping sediment microbial community. Indeed, Chen et al. (2019) has confirmed different dispersal ability for archaea and bacteria. They observed a significant decay of community similarity with the vertical spatial distance for the archaeal, bacterial and fungal communities in soil habitats. However, the slopes of their vertical spatial decay curves were steepest for archaea, followed by fungi and bacteria, indicating that archaea showed strongest Distance-Decay and weakest dispersal [25]. Our neutral community model analysis also found that bacteria and archaea had different migration rate (*m*) or dispersal abilities (Fig. 1a). Second, the mangrove sediment microbial communities can be structured by abiotic conditions such as soil pH, carbon content, etc. Indeed, exotic *S. alterniflora* has strong effects on soil conditions. For example, they can provide organic matter through leaf-litter inputs or through the release of root exudates into the soil environment, the quantity, quality and timing of litter production also changed after plant invasion [52]. Litter quality is one of the most important factors affecting soil biota, as soil fauna are more abundant when litter decomposes faster. Study has showed that invasive plants generally produce more litter than natives [53]. Mangrove sediments’ microbial communities had different tolerance to environmental conditions, thus changed the contribution of determinism. Furthermore, we found that the effects of *S. alterniflora* on the assembly processes and co-occurrence patterns of microbial communities varied between different microbial (archaea and bacteria) types (Figs. 1, 2, 5), which could be attributed to that bacteria and archaea have different adaptability to plant types and environmental changes [54]. Third, our study found that *S. alterniflora* invasion changed microbial co-occurrence patterns (Fig. 5, Table 1). Biotic interactions are crucial trait that influences the relative importance of determinism and stochasticity, thus the change of microbial co-occurrence patterns could lead to the variation of ecological processes.

The *S. alterniflora* invasion changed microbial network complexity and robustness (Fig. 5, Figs. S8-S10, Table 1, Tables S3-S4), indicating that exotic plants invasion affected the stability of the microbial community and ecosystem. The major reason could be attributed to the influence of root litter identity. *S. alterniflora* caused changes of litter identity and soil properties (i.e. organic matter), thus altered ecological networks [41]. Different plant types contained specific content of soil litter. Invasive plants could affect soil food webs through various resource inputs including belowground resource (living root-derived and root litter) and aboveground resource (shoot litter) [55]. The litter provides energy and food sources for the soil microbial community and the amount of resources usually determines the complexity and stability of the soil detritus-based food web. Litter additions from invasive plants can increase habitat heterogeneity by providing more foraging choices and shelter for soil biota, which may favour some groups of soil organisms [55]. In particular, both macrobiological and microbiological studies have shown that resource and food availability are important drivers of social network structures [56]. For example, elevated CO_2_ has been shown to increase the phylogenetic and functional complexity of microbial networks in soil, which was likely due to the increased amount of C input into soil under elevated CO_2_ [44]. Therefore, the change of soil microbial food web caused the variation of biological interactions and co-existence patterns (e.g., competition, facilitation, mutualisms, and predation). Furthermore, the network complexity and robustness analysis (Fig. S10) showed that *S. alterniflora* had different effects on archaeal and bacterial co-existence patterns, indicating that these two groups had distinct responses to change in soil environmental conditions and resource/food availability.

The keystone taxa were also varied after *S. alterniflora* invasion (Fig. S8-S9, Table S3-S4), suggesting environmental conditions determine keystone taxa. This result supported the context dependency theory that keystone taxa play critical roles only under certain conditions [57], and it also indicated that *S. alterniflora* affected the stability of microbial co-occurrence network, thus influenced mangrove ecosystem. For example, in the archaea, the Woesearchaeia occupied a large proportion of keystone species in native habitat (20 OTUs with 71% percent in mangrove vegetation type), whereas in the invaded cordgrass habitat, Bathyarchaeia occupied 9 OTUs with 69% percent in all keystone taxa (Table S3). As a dominant and newly proposed archaeal phylum, Bathyarchaeia leads both autotrophic and heterotrophic lifestyles, including the Wood-Ljungdahl pathway, acetate production, methane metabolism, and degradation of proteins and aromatic compounds [47, 58], and is believed to play an important role in global carbon cycling. Previous study showed that total organic carbon (TOC) and nitric oxide were significantly correlated with the abundance of Bathyarchaeia, suggesting that these species preferentially dwelled in slightly acidic, high TOC, and subsurface environments [59]. The *S. alterniflora* invasion altered keystone taxa and would potentially influenced mangrove ecosystem geochemical cycle. Furthermore, in the bacterial keystone species, the number of Alphaproteobacteria changed from native species (1 OTU in mangrove vegetation type) to non-native species (3 OTUs in cordgrass vegetation type) (Table S4). In subtropical mangrove ecosystems, soil denitrification has been regarded as the main source of N_2_O. Alphaproteobacteria is characterized as denitrifiers, as they can produce N_2_O [60]. These results implied that the denitrifier community was changed after the *S. alterniflora* invasion. The change of these taxa may influence other microorganisms via the network interactions, resulting in variation of microbial community composition and function. Another study also showed that the *S. alterniflora* invasion significantly increased both the abundance and diversity of denitrifiers [61]. These changes may account for the high level of mangrove sediment denitrification after the *S. alterniflora* invasion. Our study considers that the prevention and control of *S. alterniflora* invasion is important for mangrove ecosystem function and service.

## Conclusions and implications

This study provides a novel insight of ecological processes and co-occurrence relationships of the mangrove archaeal and bacterial communities under *S. alterniflora* invasion. We found that stochastic processes shaped overall archaeal and bacterial communities, and bacteria were more controlled by stochasticity than archaea. Compared to archaea, bacteria had higher dispersal ability, thus caused wider niche breadth and diversity. Co-occurrence network analysis revealed that network structure of bacteria was more complex than that of archaea. The keystone taxa mainly belonged to conditionally rare taxa, indicating they may play central roles in maintaining the stability of microbial community and ecological function. Importantly, we found that *S. alterniflora* invasion changed the relative contribution of determinism and stochasticity in shaping microbial communities assembly. And invasion showed different effects on the archaeal and bacterial networks since invasion increased archaeal network complexity and stability, but decreased the network complexity and stability of bacteria. Our study confirmed *S. alterniflora* invasion changed composition and stability of the microbial community, thus its control is important for mangrove ecosystem.

## Materials

### Study area and sediment sampling

This study was carried out in the mangrove area (117°24′ -119°7′E, 23°55′ -25°05′N) across coastline in Fujian province, southeast China. Here, 4 representative mangrove regions invaded by *S. alterniflora* were selected along latitude gradients including Zhangjiang Estuary (5 sites), Jiulong Estuary (3 sites), Quanzhou Bay (2 sites) and Meizhou Bay (2 sites) from south to north in July to August 2018. Each site including 4 different types of vegetation zones: mangrove (native mangrove zone), ecotone (ecotone area with *S. alterniflora* and mangrove growing mixed together in the same area), cordgrass (cordgrass invaded zone with *S. alterflora*), and mudflat (unvegetated bare mudflat). Finally, we collected 48 sediment samples from 12 sites at 4 mangrove regions. Since *Kandelia candel* is the most common mangrove plant in coast of southeastern China, we collected bulk mangrove sediments of *Kandelia candel* plants. All samples were collected from the top 0–10 cm layer in sediment using a polyvinyl chloride (PVC) pipe and transported to the laboratory immediately.

### DNA extraction, PCR and Illumina sequencing

The total genomic DNA of sediment archaeal and bacterial communities was extracted using a FastDNA spin kit (MP, Biomedicals, Santa Ana, CA, USA) following the manufacturer’s instructions. Microbial communities were profiled by targeting a region of the 16S rRNA gene for archaea and bacteria. The V3-V4 region of the archaeal 16S rRNA gene was PCR-amplified using the primers Arch519F (CAGCCGCCGCGGTAA) / Arch915R (GTGCTCCCCCGCCAATTCCT). The V4-V5 region of the bacterial 16S rRNA gene was amplified by using the primer pair 515F (5′ -GTG CCA GCM GCC GCG GTA A-3′) / 907R (5′ -CCG TCA ATT CCT TTG AGT TT-3′) [16]. Gene amplification was conducted in a 20-μL reaction system containing 4 μL of FastPfu Buffer (5×), 2 μL of dNTP mix (2.5 mM), 0.8 μL of each primer (5 μM), 0.4 μL of Fastpfu polymerase, 10 ng of template DNA, and 0.2 μL of BSA. The PCR parameters were 95 °C for 3 min, followed by 35 cycles of 95 °C for 30 s, 55 °C for 30 s, and 72 °C for 45 s, with a final extension at 72 °C for 10 min. Triplicate amplifications from each sample were mixed for library preparation. Asymmetric barcode sequences were ligated to the PCR primers before amplification. Adapters were then ligated to the amplicons at both ends during library preparation with the NEXTflex™ Rapid DNA-Seq Kit. Sequencing was performed on the Illumina HiSeq2500 platform (Illumina Inc., San Diego, CA, USA).

### Bioinformatics

Paired-end reads were first merged using FLASH software and then quality filtered according to the procedure described by Caporaso et al. [62]. Chimera detection and removal was accomplished using the USEARCH tool in the UCHIME algorithm. Sequences were clustered into OTUs using UPARSE [63] with the 97% sequence similarity cut-off. Representative sequence from each OTU was aligned against the SILVA (Release 132) reference alignment using the RDP classifier [64]. Unassigned OTUs (sequence similarity to a reference sequence is < 80%) and singletons (OTUs with only one sequence) were discarded prior to further analysis. Finally, to minimize biases associated with sequencing coverage and allow for comparison of community pattern among 48 samples, the sequence data were normalized to 43,372 and 6923 sequences per sample for bacteria and archaea, respectively.

### Definition of abundant and rare taxa

The definition of abundant and rare OTUs is depended on the relative abundance following the previous study [65], with the relative abundance thresholds as 0.1% for rare taxa and 1% for abundant taxa. We classified all OTUs into six categories: 1) always abundant taxa (AAT) were defined as the OTUs with relative abundance ≥1% in all samples; 2) always rare taxa (ART) were defined as the OTUs with relative abundance < 0.1% in all samples; 3) moderate taxa (MT) were defined as OTUs with relative abundance between 0.1 and 1% in all samples; 4) conditionally rare taxa (CRT) were defined as with relative abundance below 1% in all samples and < 0.1% in some samples; 5) conditionally abundant taxa (CAT) were defined as taxa with relative abundance ≥0.1% in all samples and ≥ 1% in some samples but never rare (< 0.1%); and 6) conditionally rare and abundant taxa (CRAT) were defined as OTUs with relative abundance varying from rare (< 0.1%) to abundant (≥ 1%).

### Statistical analysis

#### Alpha-diversity analysis

All alpha-diversity analyses were conducted in the R environment (version 3.6.1) using “vegan” package [66]. The rarefaction curves were calculated and a truncated Preston log-normal distribution [67] was fitted to estimate sampling effort. The bacterial and archaeal alpha diversity indices including OTU richness and Shannon-Wiener index were calculated and Tukey’s HSD test were performed to determine their significance of differences.

#### Neutral community model

The neutral community model (NCM) was used to determine the contribution of stochastic processes to microbial community assembly by predicting the relationship between the frequency with which taxa occur and their abundance across the wider metacommunity [11, 68]. In general, the model predicts that taxa that are abundant in the metacommunity will be widespread, since they are more likely to disperse by chance among different sampling sites, whereas rare taxa are more likely to be lost in different sites due to ecological drift (i.e., the stochastic loss and replacement of individuals). The estimated migration rate (*m*) is a parameter for evaluating the probability that a random loss of an individual in a local community would be replaced by dispersal from the metacommunity, and, therefore, is a measure of dispersal limitation. Higher *m* values indicate that microbial communities are less dispersal limited [11, 68]. The parameter *R*^*2*^ represents the overall fit to the neutral model. Calculation of 95% confidence intervals around all fitting statistics were done by bootstrapping with 1000 bootstrap replicates.

#### Null model

The framework developed by Stegen et al. [69] that integrates both the phylogenetic and null model analyses, was used to determine the contribution of different ecological processes to community assembly. This approach can infer not only the relative importance of determinism and stochasticity on microbial community assembly but also the sub-processes within each category. The null model expectation was generated using 999 randomizations. The variation of both phylogenetic diversity and taxonomic diversity was measured using null model-based phylogenetic and taxonomic β-diversity metrics, namely β-nearest taxon index (βNTI) and Bray–Curtis-based Raup–Crick (RC_Bray_). A significant deviation (i.e., |βNTI| > 2) indicates the dominance of deterministic processes. βNTI < − 2 indicates significantly less phylogenetic turnover than expected (i.e., homogeneous selection) while βNTI > 2 indicates significantly more phylogenetic turnover than expected (i.e., variable selection). βNTI values falling within the range of − 2 to 2 indicate stochastic processes that include homogenizing dispersal, dispersal limitation, and “undominated fraction”. To discern these three processes, RC_Bray_ was calculated. The relative influence of homogenizing dispersal was quantified as the fraction of pairwise comparisons with |βNTI| < 2 and RC_Bray_ < − 0.95. Dispersal limitation was quantified as the fraction of pairwise comparisons with |βNTI| < 2 and RC_Bray_ > 0.95. The fractions of all pairwise comparisons with |βNTI| < 2 and |RC_Bray_| < 0.95 were used to estimate influence of “undominated” assembly, which mostly consists of drift, weak selection, weak dispersal and diversification [9, 69]. To evaluate the relative importance of deterministic processes versus stochastic processes in shaping mangrove archaeal and bacterial communities, the stochasticity/determinism ratio was calculated. Here, the percentage of determinism was calculated as the sum of homogeneous selection and variable selection, and stochasticity’s percentage was calculated as the sum of dispersal limitation, homogeneous dispersal, and undominated fraction.

#### Niche breadth

To help reveal the patterns of stochasticity/determinism and their influence on microbial communities, we estimated Levins’ niche breadth (B) index [70] for the microbial group’s members according to the formula:

$$ {B}_j=1/{\sum}_{i=1}^N{P}_{ij}^2 $$

Where *B*_*j*_ is the niche breadth of OTU *j* in a metacommunity (species with high or low *B* values are referred to as habitat generalists or specialists, respectively); *N* is the total number of communities in each metacommunity; *P*_*ij*_ is the proportion of OTU *j* in community *i* [31]. A high *B*-value for a given OTU indicates its wide habitat niche breadth. The community level *B*-value (*Bcom*) was calculated as the average of *B* values from all taxa occurring in one community. We expect a microbial group with a wider niche breadth to be more metabolically flexible at the community level [28, 31]. The analysis was conducted using the “niche.width” function in “spaa” package in R [71]. To identify statistical differences of the overall difference in the *Bcom* values for bacteria and archaea, Tukey’s HSD test of archaeal and bacterial communities were conducted among four different types of vegetation zones.

#### Network analysis

We analyzed bacterial and archaeal networks for total communities and four different habitats (mangrove, ecotone, cordgrass, and mudflat) subcommunities separately. To simplify the networks for a better visualization, we removed OTUs occurring in less than 50% of all samples and kept the 300 most abundant archaeal and bacterial OTUs in the analysis. Robust correlations with Spearman’s correlation coefficients (*ρ*) > 0.6 and false discovery rate-corrected (FDR-corrected) *p*-values < 0.01 were used to construct networks using the “picante” R package [72]. Each node represents one OTU, and each edge represents a strong and significant correlation between two nodes. Node-level topological properties (degree, betweenness centrality, closeness centrality, and eigenvector centrality) were further calculated in the “igraph” R package [73]. Modules are sub-units or communities, which are sets of highly inter-connected nodes, and the rate of intra-module edges is higher than that of inter-module ones. Degree centrality is the number of paths that connect the local node to other nodes (e.g., connections between taxa); betweenness centrality refers to the potential influence of a particular node on the connections of other nodes; closeness centrality is the average distance of a node to any other node [20]. Statistical differences in measured node-level attributes across different taxa were determined using Tukey’s HSD test. Sub-network analyses of archaeal and bacterial communities were performed separately using the “igraph” package in R. Networks were visualized using the interactive Gephi 0.9.2 platform [74].

The natural connectivity provides sensitive discrimination of network structural robustness, we estimated network stability by removing nodes in the static network to assess how quickly robustness degraded and assessed network robustness by natural connectivity [75]. Further, 1000 Erdös–Réyni random networks, which had the identical number of nodes and edges as the real networks, were generated in the “igraph” R package, with each edge having the same probability of being assigned to any node [76]. Topology characteristics of both real and random networks were calculated and compared, including modularity, clustering coefficient and average path length. By determining the most interacted microbial taxa, networks can also be used to identify keystone species. A Zi-Pi plot was used to identify key populations based on the nodes’ roles in their own network Zi indicates how well a node connects to nodes within the same module, while Pi indicates how well a node connects to other modules. Based on within-module and among-module connectivity, topological roles of different nodes were divided into four categories, (i) network hubs: nodes with Zi > 2.5 and Pi > 0.62; (ii) module hubs: nodes with Zi > 2.5 and Pi ≤0.62; (iii) connectors: nodes with Zi ≤ 2.5 and Pi > 0.62; and (iv) peripheral nodes: nodes with Zi ≤ 2.5 and Pi ≤0.62. Network hubs, module hubs, and connectors were regarded keystone taxa, which are considered to play important roles in the microbial community structure and potential functions [23].

## Supplementary Information

**Additional file 1: Fig. S1.** Sketch map of Fujian coastal mangrove sediments showing the sampling sites. 4 representative mangrove regions invaded by *S. alterniflora* were selected along latitude gradients including Zhangjiang Estuary (5 sites), Jiulong Estuary (3 sites), Quanzhou Bay (2 sites) and Meizhou Bay (2 sites) from south to north in July to August 2018. Each site including 4 different types of vegetation zones: mangrove (native mangrove zone), ecotone (ecotone area with *S. alterniflora* and mangrove growing mixed together in the same area), cordgrass (cordgrass invaded zone with *S. alterflora*), and mudflat (unvegetated bare mudflat). Finally, we collected 48 sediment samples from 12 sites at 4 mangrove regions. The map was performed using ArcGIS 10.1 (ESRI, Redlands, CA, USA). **Fig. S2.** Archaeal and bacterial diversity of mangrove sediment. **A:** Rarefaction curves of similarity-based operational taxonomic unit (OTU) at 97% sequence similarity level of 48 samples. **B:** OTU abundance distribution and fit to the Preston log-normal model using two approximations: maximized likelihood to log_2_ abundances (blue line) and Quasi-Poisson fit to octaves (red line). Calculation of the Preston veil, which infers the number of OTUs that we missed during our sampling, confirmed that we captured most of the archaeal and bacteria richness, thus allowing extraction of general patterns of archaeal and bacteria biodiversity from our data set. **Fig. S3.** Comparison of richness and Shannon-Wiener index between overall archaeal and bacterial communities. ***, *P* < 0.01 (Tukey’s HSD test). **Fig. S4.** Comparison of richness and Shannon-Wiener index among four different types of vegetation zones of archaeal and bacterial communities. No significant differences were found among different vegetation zones of archaeal and bacterial richness and Shannon-Wiener index based on Tukey’s HSD test. **Fig. S5.** Relative abundance of archaeal and bacterial taxa at phylum level among four different types of vegetation zones. **Fig. S6.** The network degree distribution patterns of archaea and bacteria. **Fig. S7.** Comparison of node-level topological features among four different types of vegetation zones of archaeal and bacterial subcommunities. The top and bottom boundaries of each box indicate the 75th and 25th quartile values, respectively, and lines within each box represent the median values. Different letters indicate the significant level at *P* < 0.01 level determined by Tukey’s HSD test. **Fig. S8.** Zi-Pi plot showing the distribution of archaeal OTUs among four different types of vegetation zones based on their topological roles. Each symbol represents an OTU. The topological role of each OTU was determined according to the scatter plot of within-module connectivity (Zi) and among-module connectivity (Pi). **Fig. S9.** Zi-Pi plot showing the distribution of bacterial OTUs among four different types of vegetation zones based on their topological roles. Each symbol represents an OTU. The topological role of each OTU was determined according to the scatter plot of within-module connectivity (Zi) and among-module connectivity (Pi). **Fig. S10.** Network robustness analysis of archaeal and bacterial communities among four different types of vegetation zones in the mangrove sediments.

**Additional file 2: Supplementary Table S1.** The contribution of each taxa category to the archaea and bacteria community in the 48 samples at 97% identity level. **Supplementary Table S2.** Lists of keystone taxa in co-occurrence network of archaea and bacteria. **Supplementary Table S3.** Lists of keystone taxa in co-occurrence network of archaea among four different types of vegetation zones. **Supplementary Table S4.** Lists of keystone taxa in co-occurrence network of bacteria among four different types of vegetation zones.

## Data Availability

The raw sequence data reported in this paper are available in the NCBI Sequence Read Archive under BioProject PRJNA656114 the accession number SRP122256.
