# Supplementary material for: Archaeal and bacterial communities assembly and co-occurrence networks in subtropical mangrove sediments under Spartina alterniflora invasion
Source: Environ Microbiome. 2021 May 3;16:10. doi: 10.1186/s40793-021-00377-y (PMC8091715; doi:10.1186/s40793-021-00377-y)
Supplement: Supplementary file 1 — Additional file 1: Fig. S1. Sketch map of Fujian coastal mangrove sediments showing the sampling sites. 4 representative mangrove regions invaded by S. alterniflora were selected along latitude gradients including Zhangjiang Estuary (5 sites), Jiulong Estuary (3 sites), Quanzhou Bay (2 sites) and Meizhou Bay (2 sites) from south to north in July to August 2018. Each site including 4 different types of vegetation zones: mangrove (native mangrove zone), ecotone (ecotone area with S. alterniflora and mangrove growing mixed together in the same area), cordgrass (cordgrass invaded zone with S. alterflora), and mudflat (unvegetated bare mudflat). Finally, we collected 48 sediment samples from 12 sites at 4 mangrove regions. The map was performed using ArcGIS 10.1 (ESRI, Redlands, CA, USA). Fig. S2. Archaeal and bacterial diversity of mangrove sediment. A: Rarefaction curves of similarity-based operational taxonomic unit (OTU) at 97% sequence similarity level of 48 samples. B: OTU abundance distribution and fit to the Preston log-normal model using two approximations: maximized likelihood to log2 abundances (blue line) and Quasi-Poisson fit to octaves (red line). Calculation of the Preston veil, which infers the number of OTUs that we missed during our sampling, confirmed that we captured most of the archaeal and bacteria richness, thus allowing extraction of general patterns of archaeal and bacteria biodiversity from our data set. Fig. S3. Comparison of richness and Shannon-Wiener index between overall archaeal and bacterial communities. ***, P < 0.01 (Tukey’s HSD test). Fig. S4. Comparison of richness and Shannon-Wiener index among four different types of vegetation zones of archaeal and bacterial communities. No significant differences were found among different vegetation zones of archaeal and bacterial richness and Shannon-Wiener index based on Tukey’s HSD test. Fig. S5. Relative abundance of archaeal and bacterial taxa at phylum level among four different types of ve [file 40793_2021_377_MOESM1_ESM.docx]

*Supplementary information of the article:*

**Archaeal and bacterial communities assembly and co-occurrence networks in subtropical mangrove sediments under *Spartina alterniflora* invasion**

Weidong Chen and Donghui Wen*

College of Environmental Sciences and Engineering, Peking University, Beijing, 100871, China

**Running title:** Mangrove microbial community assembly and network

**Keywords:** Community assembly / Co-occurrence network / Bacterial community / Archaeal community / Mangrove / *Spartina alterniflora*

*** Correspondence:**

1. mail: [dhwen@pku.edu.cn](mailto:dhwen@pku.edu.cn); Tel & Fax: +86 10 62751923

**This additional information contains:**

- - 11 Pages
  - 10 Figures

**
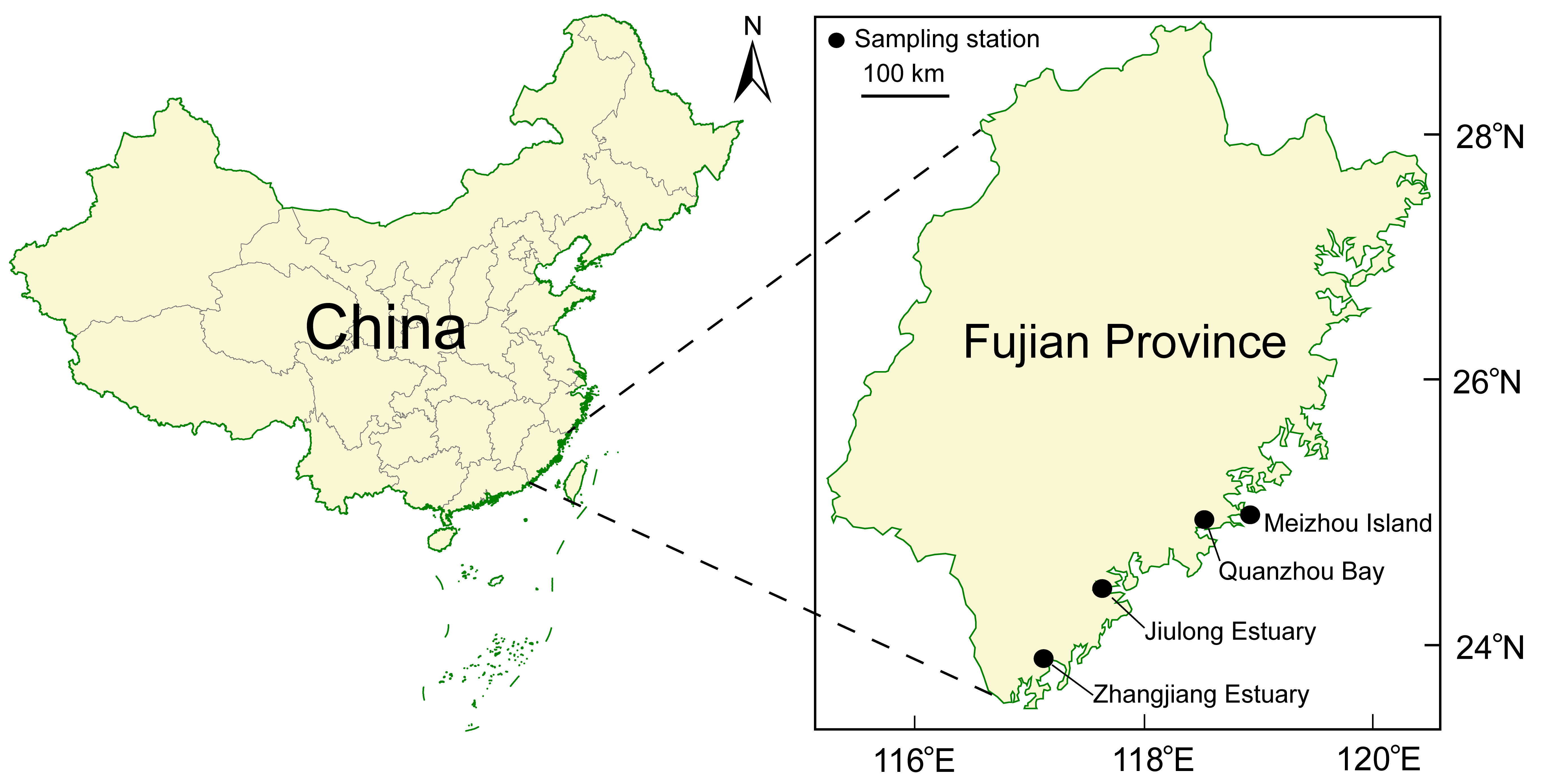
**

**Fig. S1** Sketch map of Fujian coastal mangrove sediments showing the sampling sites. 4 representative mangrove regions invaded by *S. alterniflora* were selected along latitude gradients including Zhangjiang Estuary (5 sites), Jiulong Estuary (3 sites), Quanzhou Bay (2 sites) and Meizhou Bay (2 sites) from south to north in July to August 2018. Each site including 4 different types of vegetation zones: mangrove (native mangrove zone), ecotone (ecotone area with *S. alterniflora* and mangrove growing mixed together in the same area), cordgrass (cordgrass invaded zone with *S. alterflora*), and mudflat (unvegetated bare mudflat). Finally, we collected 48 sediment samples from 12 sites at 4 mangrove regions. The map was performed using ArcGIS 10.1 (ESRI, Redlands, CA, USA).

**
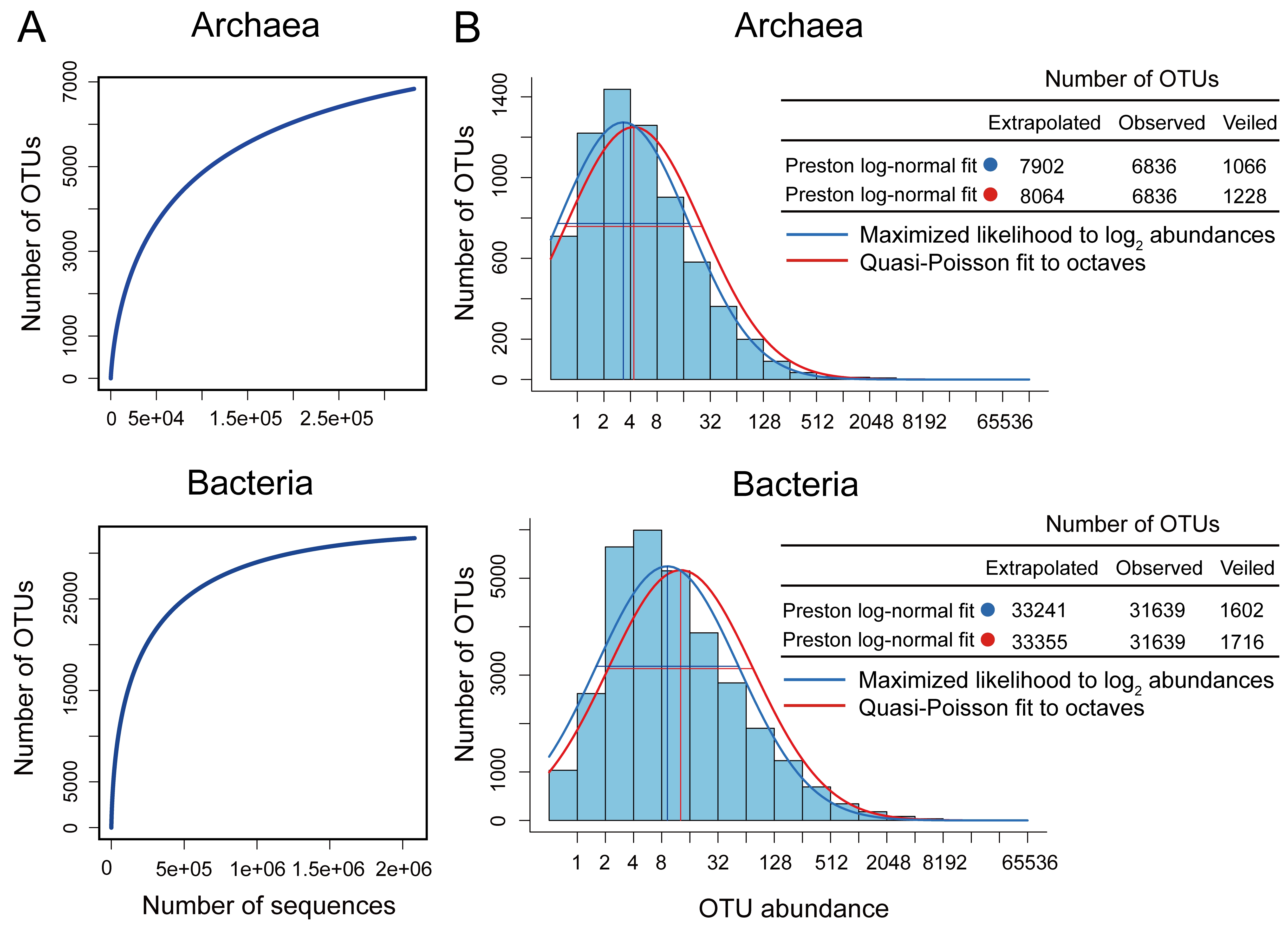
**

**Fig. S2** Archaeal and bacterial diversity of mangrove sediment. **A:** Rarefaction curves of similarity-based operational taxonomic unit (OTU) at 97% sequence similarity level of 48 samples. **B:** OTU abundance distribution and fit to the Preston log-normal model using two approximations: maximized likelihood to log_2_ abundances (blue line) and Quasi-Poisson fit to octaves (red line). Calculation of the Preston veil, which infers the number of OTUs that we missed during our sampling, confirmed that we captured most of the archaeal and bacteria richness, thus allowing extraction of general patterns of archaeal and bacteria biodiversity from our data set.


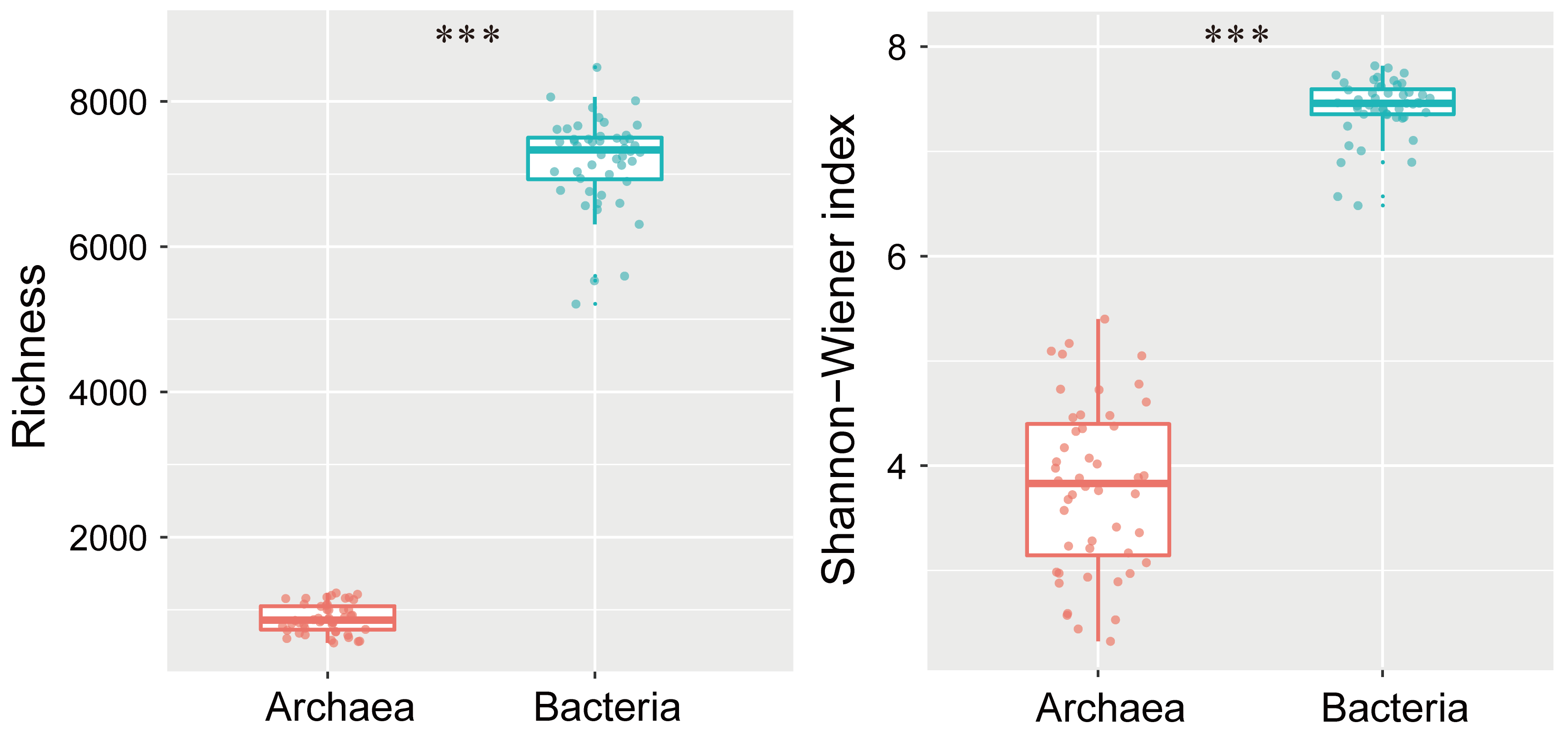


**Fig. S3** Comparison of richness and Shannon-Wiener index between overall archaeal and bacterial communities. ***, *P* < 0.01 (Tukey’s HSD test).


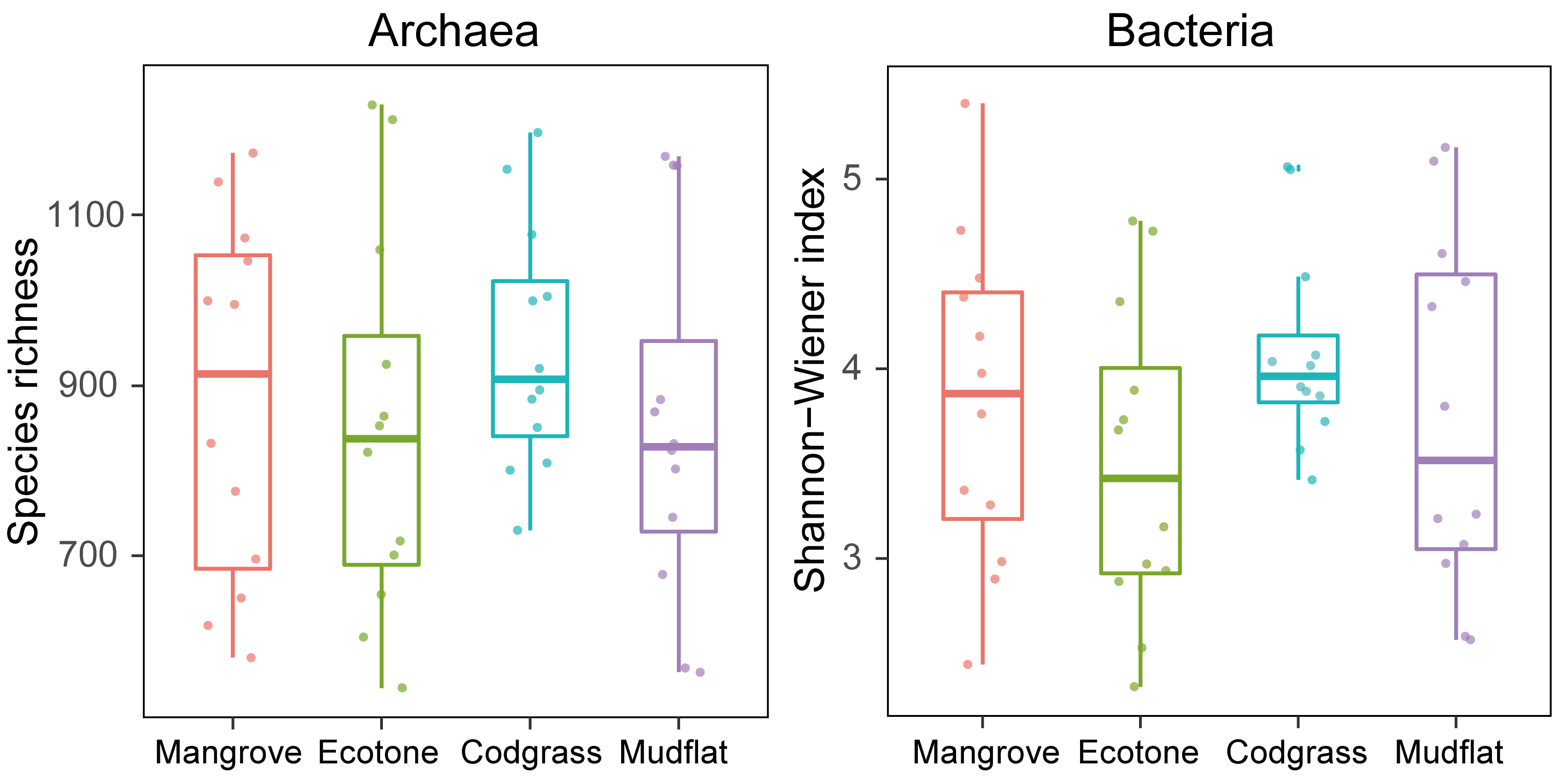


**Fig. S4** Comparison of richness and Shannon-Wiener index among four different types of vegetation zones of archaeal and bacterial communities. No significant differences were found among different vegetation zones of archaeal and bacterial richness and Shannon-Wiener index based on Tukey’s HSD test.


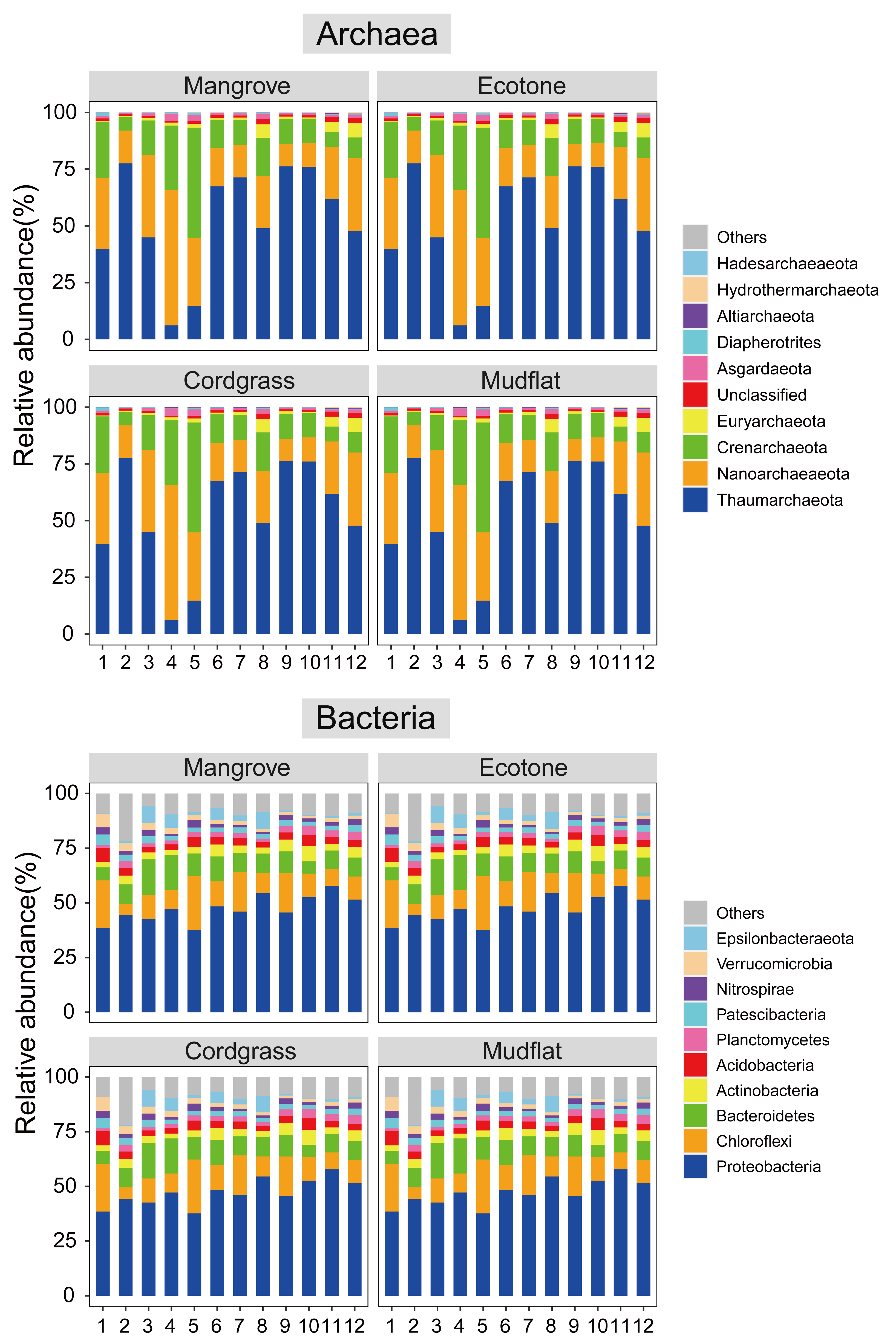


**Fig. S5** Relative abundance of archaeal and bacterial taxa at phylum level among four different types of vegetation zones.


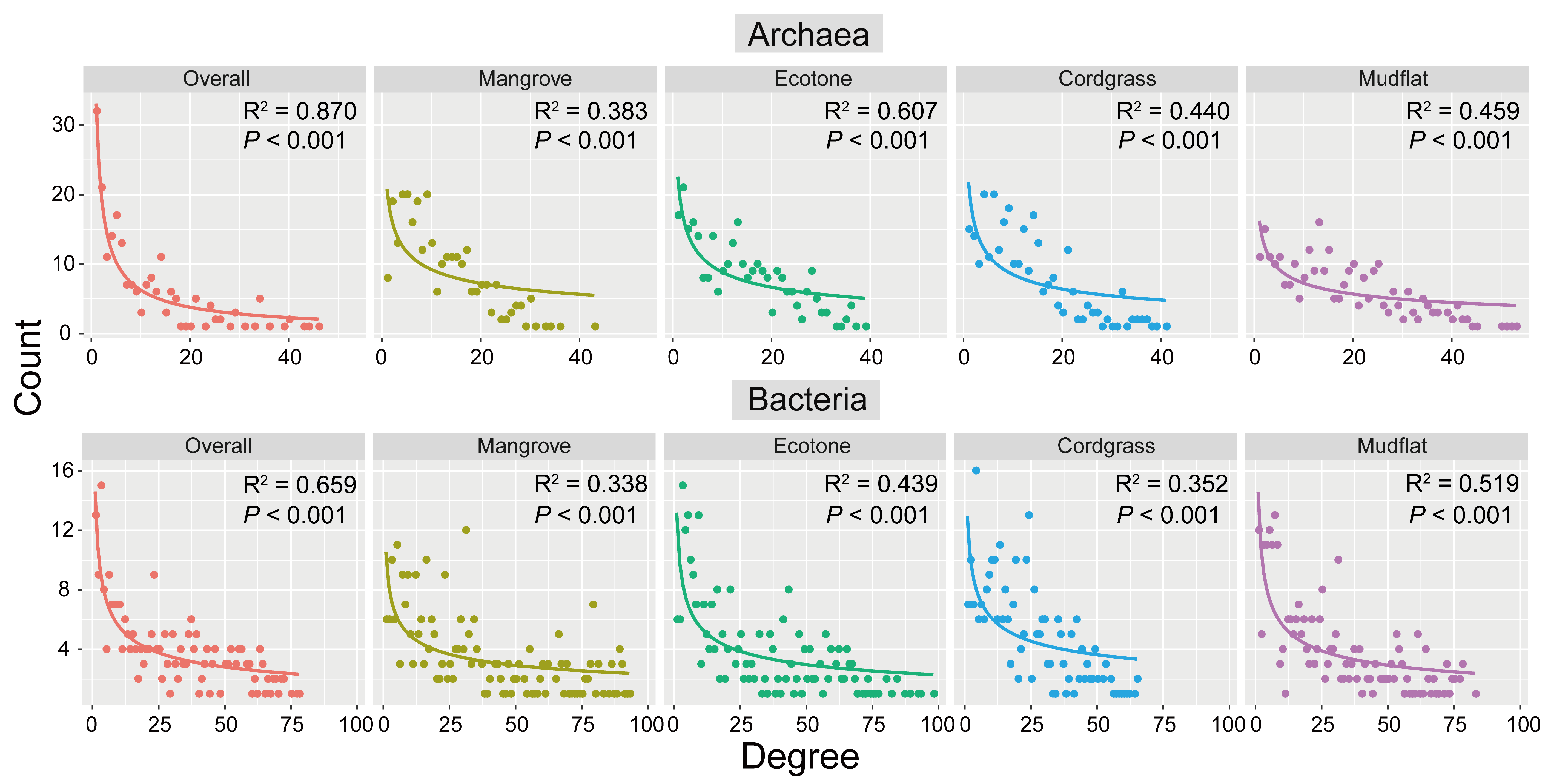


**Fig. S6** The network degree distribution patterns of archaea and bacteria.


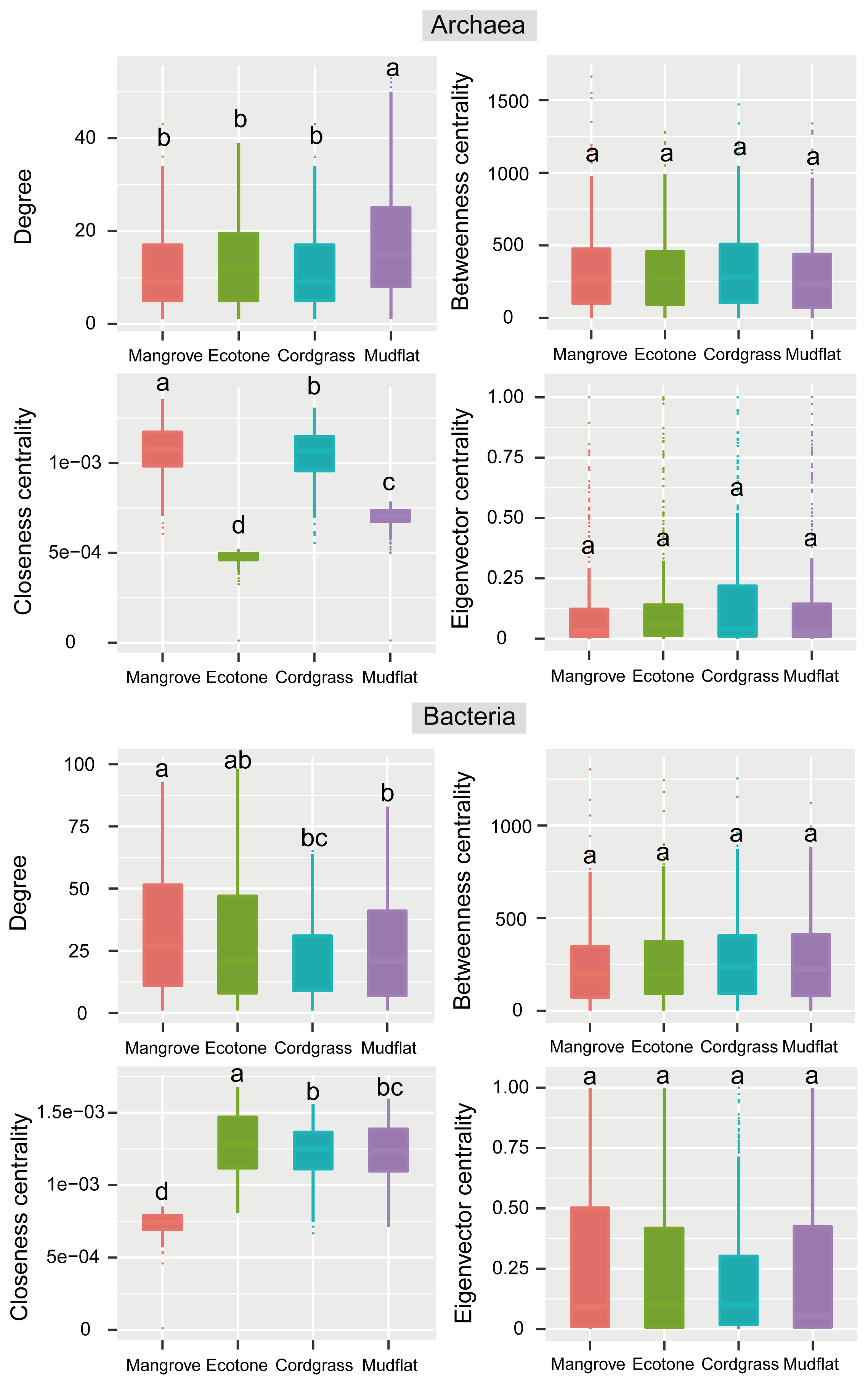


**Fig. S7** Comparison of node-level topological features among four different types of vegetation zones of archaeal and bacterial subcommunities. The top and bottom boundaries of each box indicate the 75^th^ and 25^th^ quartile values, respectively, and lines within each box represent the median values. Different letters indicate the significant level at *P* < 0.01 level determined by Tukey’s HSD test.


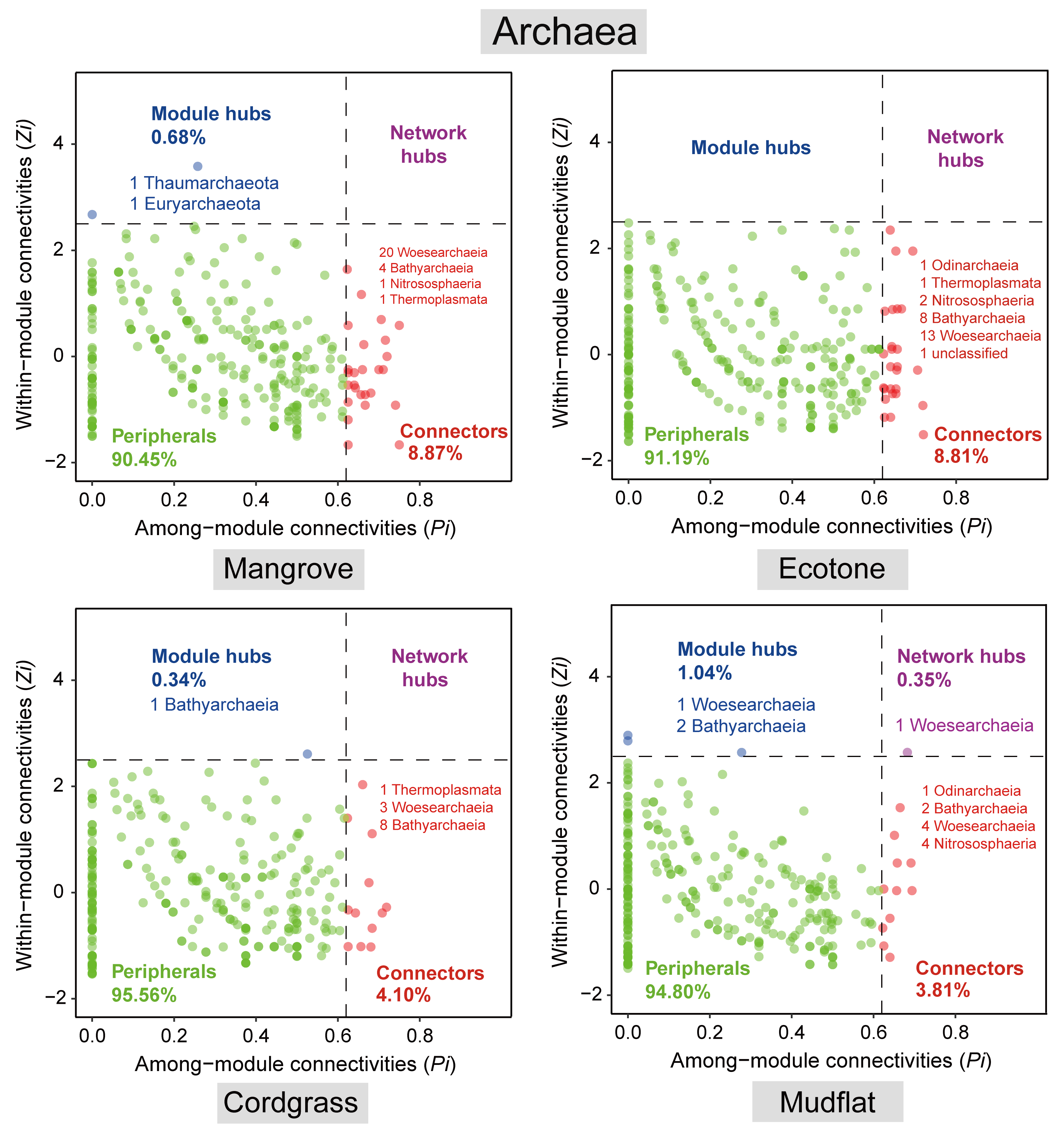


**Fig. S8** Zi-Pi plot showing the distribution of archaeal OTUs among four different types of vegetation zones based on their topological roles. Each symbol represents an OTU. The topological role of each OTU was determined according to the scatter plot of within-module connectivity (Zi) and among-module connectivity (Pi).


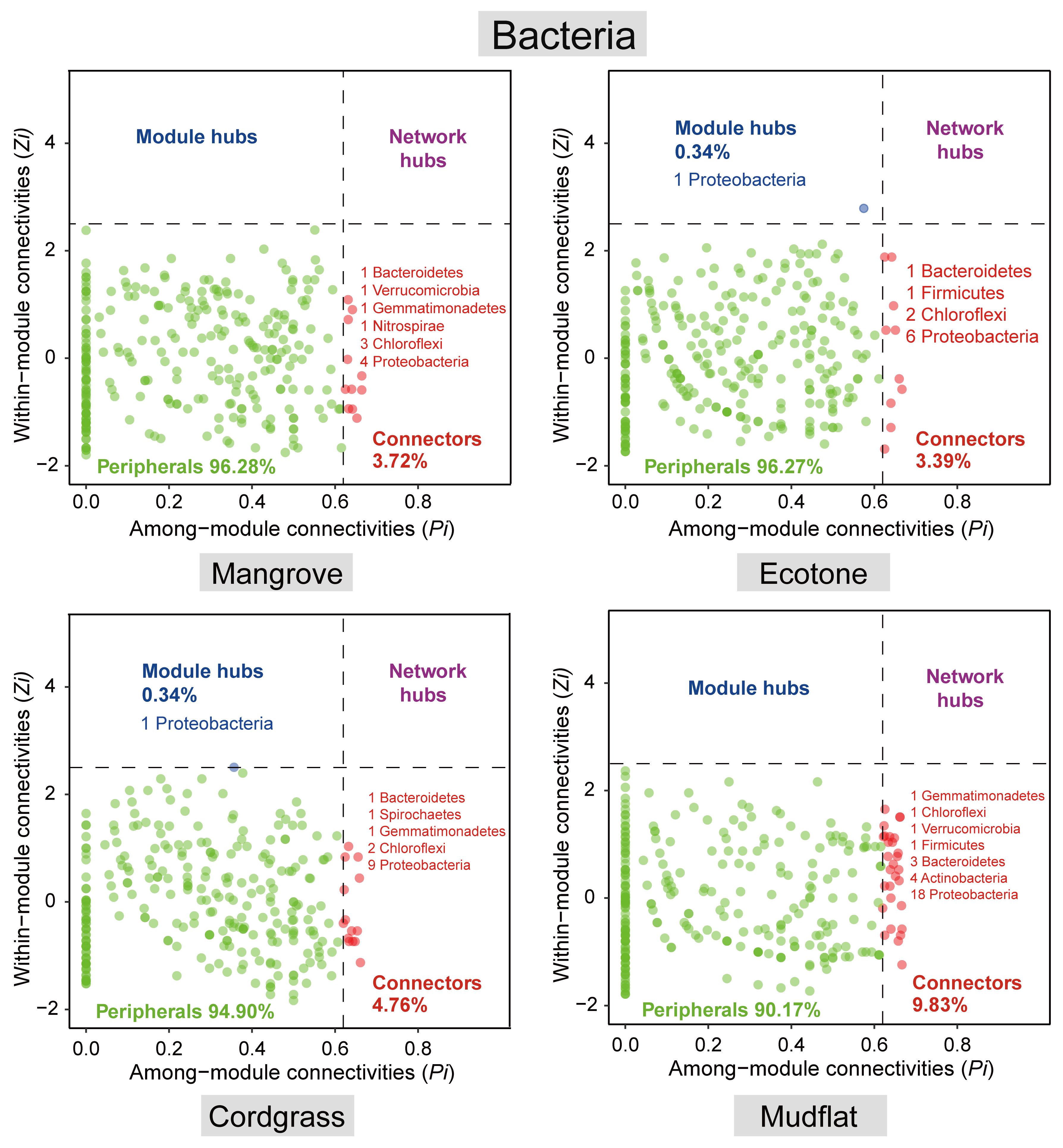


**Fig. S9** Zi-Pi plot showing the distribution of bacterial OTUs among four different types of vegetation zones based on their topological roles. Each symbol represents an OTU. The topological role of each OTU was determined according to the scatter plot of within-module connectivity (Zi) and among-module connectivity (Pi).


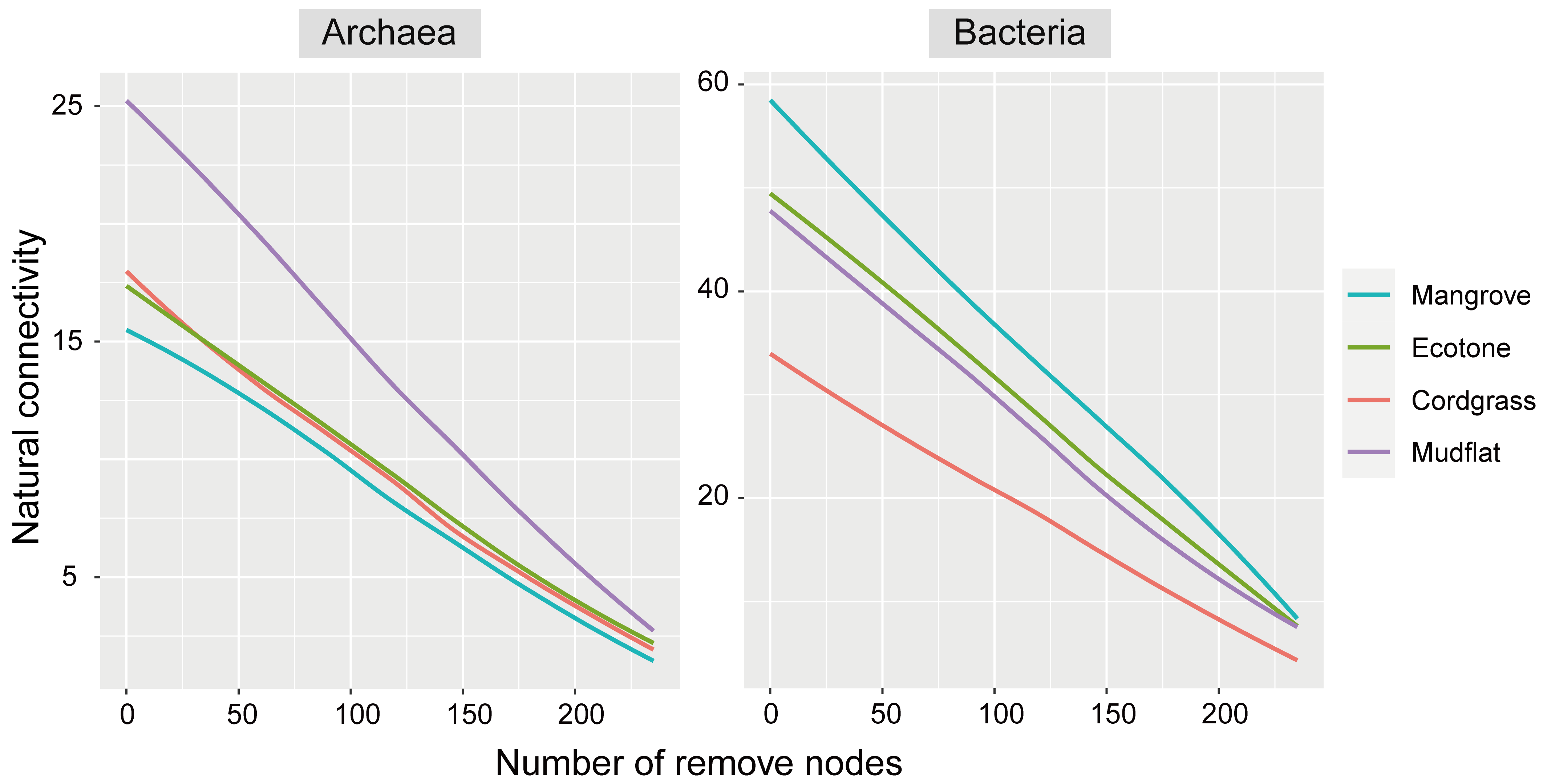


**Fig. S10** Network robustness analysis of archaeal and bacterial communities among four different types of vegetation zones in the mangrove sediments.
